# Unveiling the health consequences of air pollution in the world’s most polluted nations

**DOI:** 10.1038/s41598-024-60786-0

**Published:** 2024-04-29

**Authors:** Mohammad Naim Azimi, Mohammad Mafizur Rahman

**Affiliations:** School of Business, University of Southern Queensland, Toowoomba, QLD 4350 Australia

**Keywords:** Air pollution, PM_2.5_, Life expectancy, Infant mortality rates, Institutional quality, Ecology, Health care

## Abstract

Air pollution poses a persuasive threat to global health, demonstrating widespread detrimental effects on populations worldwide. Exposure to pollutants, notably particulate matter with a diameter of 2.5 µm (PM_2.5_), has been unequivocally linked to a spectrum of adverse health outcomes. A nuanced understanding of the relationship between them is crucial for implementing effective policies. This study employs a comprehensive investigation, utilizing the extended health production function framework alongside the system generalized method of moments (SGMM) technique, to scrutinize the interplay between air pollution and health outcomes. Focusing on a panel of the top twenty polluted nations from 2000 to 2021, the findings yield substantial insights. Notably, PM_2.5_ concentration emerges as a significant factor, correlating with a reduction in life expectancy by 3.69 years and an increase in infant mortality rates by 0.294%. Urbanization is found to increase life expectancy by 0.083 years while concurrently decreasing infant mortality rates by 0.00022%. An increase in real per capita gross domestic product corresponds with an improvement in life expectancy by 0.21 years and a decrease in infant mortality rates by 0.00065%. Similarly, an elevated school enrollment rate is associated with a rise in life expectancy by 0.17 years and a decline in infant mortality rates by 0.00032%. However, a higher population growth rate is found to modestly decrease life expectancy by 0.019 years and slightly elevate infant mortality rates by 0.000016%. The analysis reveals that per capita greenhouse gas emissions exert a negative impact, diminishing life expectancy by 0.486 years and elevating infant mortality rates by 0.00061%, while per capita energy consumption marginally reduces life expectancy by 0.026 years and increases infant mortality rates by 0.00004%. Additionally, economic volatility shock presents a notable decrement in life expectancy by 0.041 years and an increase in infant mortality rates by 0.000045%, with inflationary shock further exacerbating adverse health outcomes by lowering life expectancy by 0.70 years and elevating infant mortality rates by 0.00025%. Moreover, the study scrutinizes the role of institutional quality, revealing a constructive impact on health outcomes. Specifically, the institutional quality index is associated with an increase in life expectancy by 0.66% and a decrease in infant mortality rates by 0.0006%. Extending the analysis to examine the nuanced dimensions of institutional quality, the findings discern that economic institutions wield a notably stronger positive influence on health outcomes compared to political and institutional governance indices. Finally, the results underscore the pivotal moderating role of institutional quality in mitigating the deleterious impact of PM_2.5_ concentration on health outcomes, counterbalancing the influence of external shocks, and improving the relationships between explanatory variables and health outcome indicators. These findings offer critical insights for guiding evidence-based policy implications, with a focus on fostering resilient, sustainable, and health-conscious societies.

## Introduction

**Figure 1:**
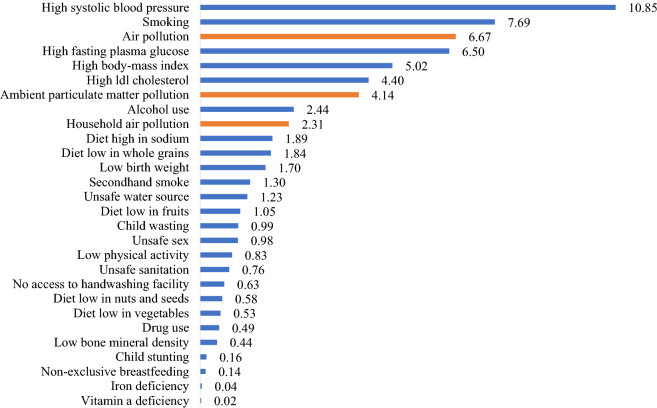
Number of deaths by health risk factors. Notes: Values are in millions of people. Source: Global burden of disease^6^. The plot has been created by authors.

Air pollution is a significant concern in the contemporary world, serving as a primary contributor to premature deaths and severe health conditions. It poses substantial risks to both public health and the natural environment, ranging from household-level smoke emissions to citywide atmospheric haze. Air pollution entails the alteration of natural features of in indoor or ambient environment due to the release of biological, chemical, or physical substances^1^. Major contributors to air pollution encompasses substandard vehicles using polluting fuels, unsustainable household practices highly involving the use of carbon-emitting fuels and substances for heating, cooking, and lighting, coal consumption, and the unregulated burning of waste materials. According to the World Health Organization’s (WHO) report^2^, the global population faces a severe risk of air pollution, with approximately 99% of people breathing air containing significant pollutant substances that surpass the recommended threshold levels set by the WHO. Figure 1 illustrates the distribution of deaths attributable to different risk factors. Air pollution holds the third position as a risk factor, following high blood pressure (first) and smoking (second). Air pollution was a contributing cause of death in 2019, with over 6.67 million deaths globally, of which 4.14 million were attributed to ambient pollution and 2.31 million to household pollution. Air pollution annually contributes to an extensive range of chronic, respiratory, and cardiovascular diseases^3,4^. Moreover, long-term exposure to air pollution is perceived to have determinantal effects on life expectancy. Existing evidence indicates that, on average, air pollution has led to a global reduction in life expectancy by one year and eight months^5^.

Studies show that lower-middle- and low-income countries are highly vulnerable to the health risk of air pollution compared to their high-income counterparts^7,8^. This vulnerability is primarily attributed to the dual negative impact of air pollution, stemming from inefficient transportation modes, substandard heating systems, increased energy and fossil fuel consumption, as well as the release of pollutants such as dirt, dust, smoke, soot, liquid droplets, and industrial chemicals into the atmosphere^9,10^. The ambient pollution resulting from these substance constitute particulate matter (PM) and is considered as a primary source of air pollution^11^. Particles with a diameter of $\le 2.5$ micrometers (PM_2.5_) are identified as posing the greatest risks to people’s health outcomes. Specifically, exposure to PM_2.5_ increase the likelihood of cardiovascular diseases, asthma, reduced visibility, and others adverse effects^12–14^, leading to a higher rate of premature mortality and a reduction in life expectancy^15^. Apte et al.^16^ investigated the impact of PM_2.5_ on life expectancy at birth, revealing that highly polluted countries, particularly in Asia and Africa, saw a reduction of 1.2 to 1.9 years of life expectancy at birth in 2016, surpassing the global average reduction of 1 year.

**Figure 2:**
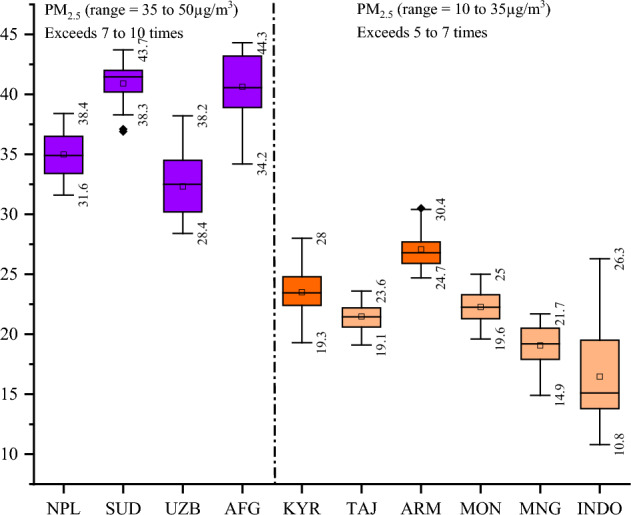
PM_2.5_ concentration 2000 to 2021. Notes: NPL: Nepal, SUD: Sudan, AFG: Afghanistan, UZB: Uzbekistan, KYR: Kyrgyzstan, TAJ: Tajikistan, ARM: Armenia, MON: Montenegro, MNG: Mongolia, INDO: Indonesia. Source: Atmospheric Composition Analysis Group of Washington University. The plot has been created by authors.

While several studies have explored the relationship between PM_2.5_ and health outcomes across different countries, considering the endogenous impact of pollutant predictors (specifically, PM_2.5_, PM_10_, and O_3_) on health, our extensive review highlights a significant gap in the existing literature. No studies, to the best of our knowledge, have extensively examined the explanatory power of exogenous factors influencing both the direct and modulated correlation between PM_2.5_ and health outcomes, leaving room for practical insights in this domain. Revisiting the subject from a different lens, our study primarily aims to analyze the impact of exogenous predictors on the relationship between PM_2.5_ and health outcomes. We intend to address existing policy gaps by offering a comprehensive understanding of how external factors shape this relationship. Furthermore, our goal is to contribute to a broader comprehension of the global health implications of air pollution, with a specific focus on the correlation between PM_2.5_ and health outcomes. To tackle these objectives, we frame three pivotal research questions pertinent to present context: First, what is the concurrent effect of air pollution on life expectancy at birth and the mortality rate? Second, how does WHO’s strategy to reduce air pollution through institutional arrangements gain practicality in an empirical sense and policy perspectives? Third, how could other externalities such as global shocks, wars, and political and trade tensions be well explained in relation to the contemporary air pollution catastrophe? To address these critical questions, we focus on the top 20 most polluted countries in the world—the nations that surpass the threshold level of PM_2.5_ (0–5μg/m^3^) recommended by the WHO. The selection of the geographical context is underpinned by two compelling rationales: First, the selected countries collectively represent one-third of world’s total population, amounting to 2.6 billion people. Many of them grapple with multidimensional poverty, food insecurity, subpar institutional quality, and heightened vulnerability to external global economic and environmental shocks. From a policymaking standpoint, our study offers precise insights into the subject matter, shedding light on specific areas that demand urgent and concerted policy attention. To substantiate this, Fig. 2 illustrates that PM_2.5_ concentration in Nepal, Sudan, Uzbekistan, and Afghanistan ranges from 35 to 50μg/m^3^, while it ranges from 10 to 35μg/m^3^ in Kyrgyzstan, Tajikistan, Armenia, Montenegro, Mongolia, and Indonesia, surpassing the recommended threshold level by 7 to 10 times and 5 to 7 times, respectively.

**Figure 3:**
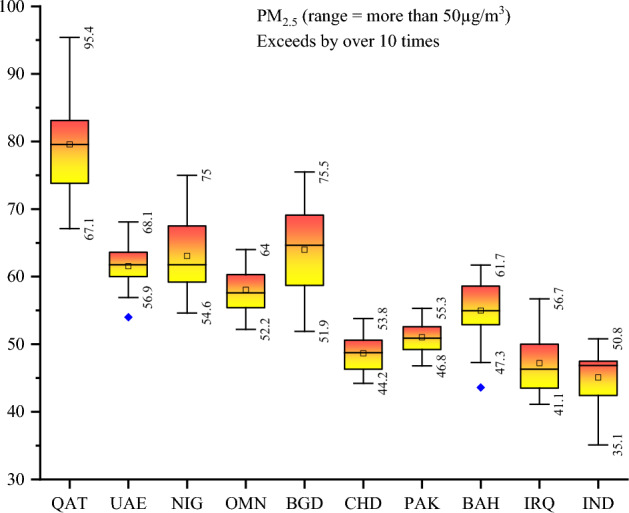
PM_2.5_ concentration 2000 to 2021. Notes: QAT: Qatar, UAE: United Arab Emirates, NIG: Nigeria, OMN: Oman, BGD: Bangladesh, CHD: Chad, PAK: Pakistan, BAH: Bahrain, IRQ: Iraq, IND: India. Source: Atmospheric Composition Analysis Group of Washington University. The plot has been created by authors.

In a more severe scenario, Fig. 3 depicts that PM_2.5_ concentration exceeds 50μg/m^3^ in Qatar, the United Arab Emirates, Nigeria, Oman, Bangladesh, Chad, Pakistan, Bahrain, Iraq, and India. Prior literature has aptly acknowledged the transboundary sources of pollution, signifying that air pollution not only impacts its immediate locality but also traverses borders, influencing other nations. This underscores the need to shift our attention to the most polluted countries. Second, while prior literature has delved into the impact of air pollution on health outcomes at the country level^17,18^, regional level^19,20^, and within economically classified regions^21^, there is a notable absence of studies considering an extensive and examination of pollutants. This gap in the literature prompted us to carry out this piece of research in the context of the top 20 most polluted countries, collectively assessing the present situation. However, the study is additionally motivated by the application of two crucial policy instruments: the role of institutional quality in elucidating the correlation between air pollution, and the influence of global economic and inflationary shocks in modulating the effects of air pollution on health. This aligns with our second research question, addressing the WHO’s strategy to mitigate the risk of air pollution through the enhancement of institutional quality. It also aligns with SDG7 (ensuring access to affordable, reliable, sustainable, and modern energy), SDG12 (responsible consumption and production), and SDG13 (taking urgent action to combat climate change and its impacts). This approach aims to precisely quantify the impact of air pollution on health by considering the variability of PM_2.5_ concentration in the presence of external shocks and existing institutional arrangements, moving beyond unconditional effects.

To our knowledge, this study represents a novel empirical investigation in the existing literature, providing unique insights from both the scope and methodological application perspectives in exploring the relationships between air pollution and the predictors of health outcomes. The contributions of this study can be summarized as follows: First, our results build upon prior findings, e.g.,^7,22,23^, and^16^ by emphasizing that pollutant-endogenous predictors are significant in explaining health outcomes, but the importance of pollutant external drivers cannot be overlooked, specifically in comprehensive and macro-level policy discussions. Second, while prior literature contributes valuably to enhancing the contemporary body of knowledge on the impact of air pollution on health outcomes, our study stands out with its nuanced policy-oriented perspective. Recognizing that no one is immune from the risk of air pollution, the study engages in a distinctive and collective empirical discussion on the most vulnerable areas where multidimensional factors, both endogenous and exogenous, drive air pollution. This allows us to draw strategic conclusions and inform appropriate policy directions. Third, by delving into critical policy discussions, the study introduces three key variables—institutional quality, inflationary shocks, and economic shocks variables—to test how these factors influence the nexus between air pollution and health outcomes. It is highly important to underline how institutional arrangements can simultaneously moderate the negative effects of air pollution on the subject and how the improvement of institutional quality can play a role in minimizing the impact of external shocks on air pollution, thus the health outcome predictors. Fourth, unlike prior evidence, this study develops a critical understanding of the complex nature of the relationship between air pollution and health outcomes through both primary and secondary approaches to exogenous variables. It precisely highlights key areas of policy tension in the contemporary period, using a contextual framework that is often unstable, poorly governed, and highly exposed to multidimensional risk factors.

## Literature review

The existing body of literature converges with our study along six distinct perspectives. The first category of sudies has focued on examining the effects of economic growth on health outcomes. For instance, Lago-Peñas et al.^24^ scrutinized the impact of per capita GDP on health expenditures in 31 OECD member countries. Their findings indicate a significant sensitivity of health expenditures to changes in per capita GDP over the long-term. Specifically, they demonstrated that an increase in per capita GDP influences health outcomes. Thoa et al.^25^ conducted a study to explore the effects of variations in healthcare utilization associated with changing economic conditions in Vietnam, using cross-sectional data from 11,260 families between 2003 and 2007. The findings revealed that out-of-pocket expenditures constitute the primary source of financing health expenditures. Additionally, economic growth was identified as a factor contributing to higher (lower) healthcare gaps between rich (poor) households. In a study by Jakovljevic et al.^26^, the authors delved into the relationship between health outcomes and per capita GDP growth in Emerging Markets Seven (EM7) and G7 countries. Their observation indicated that during periods of economic recession, every unit reduction in per capita GDP growth significantly reduced health expenditures. The authors argued that economic growth plays a key role in influencing health outcomes. Moreover, Niu et al.^27^ conducted a study investigating the effects of economic growth on public health in China, employing a panel threshold model with data spanning from 2000 to 2017. Their findings indicated a threshold effect of economic growth, suggesting that when growth surpasses a certain threshold level, there is a significant improvement in public health. On the contrary, growth below the threshold level did not show significant impact. The results were contingent on panel heterogeneity, revealing that the threshold effect of growth was valid in some provinces of China but invalid in others. Likewise, the literature reports many other studies that have explored the unconditional effects of growth on different indicators of health outcomes (*inter alia*,^28–30^), all acknowledging the positive impact of growth on health outcomes. However, this category of studies falls short in considering the gradually unfolding long-run impact of economic shocks and episodes of hyperinflationary on the subject.

The second category of studies concentrates on elucidating the effects of social factors on public health indicators. For instance, Cohen and Syme^31^ demonstrated that educational attainment serves as a critical driver of health through various conduits, including biological aging, health awareness, life chances, risk factors, and neural development. The authors underscored the importance of awareness from childhood to kindergarten and from preschool to higher education in advancing health outcomes. Similarly, Baker et al.^32^ assessed the impact of education on various health risk factors, such as drug abuse, smoking, accidents, and diseases. Their findings provided statistical evidence supporting the notion that education serves as an effective tool in enhancing health conditions by reducing the prevalence of risk factors for individuals. Furthermore, they argued that education should be recognized as an autonomous causal agent rather than a secondary contributor to the advancement of public health. Albert and Davia^33^ explored the link between education and health in developed economies, employing educational attainment and self-reported health conditions in a panel of 11 European Union member countries using a random effects probit estimation technique. Their findings revealed that secondary education has a positive impact on health status in certain regions of the panel, while educational attainment has been shown to universally improve health conditions across all included countries in their panel. Additionally, Raghupathi and Raghupathi^34^ investigated the effects of education on health using visual analytic method in 26 OECD countries from 1995 to 2015. Their findings indicated that individuals with higher levels of education tend to have a longer lifespan and better health, while those with lower levels of education experience a shorter productive lifespan and lower health status. This group of studies has acknowledged the importance of social factors on public health outcomes across different countries, though it has ignored examining the role of institutional setups in enhancing social inclusion in enhancing subject efficiency.

The effects of demographic composition, including poplution density and urbanization have been explored in relation to health outcome indictors; however, the number of these studies is still limited in the literature. The results presented by the third category of studies are mixed. For example, Torres et al.^35^ measured the impact of urbanization on life expectancy and mortality rates using a method of decomposition to split the variations in life expectancy and changes in population composition. The authors found that an uneven distribution of population in urban areas negatively impacts life expectancy and significantly increases the rate of mortality. Contrary to that, Jemiluyi^36^ evaluated the effects of urbanization on child health outcomes in Nigeria using datasets about infant, neonatal, and child mortality rates under 5 years old. The authors employed panel regression and a fully modified least squares estimation to test their data. They observed a long-run relationship between the child health outcome and urbanization and found that urbanization has a long-run negative impact on the child mortality rate. In the context of China, Shao et al. ^37^ investigated the asymmetric effects of urbanization on healthcare expenditures and noticed that higher urbanization results in increasing the overall healthcare expenditures both in the central and eastern regions of China. Further, the authors found that the aging population has a positive association with healthcare expenditures. In the same vein, Jiang et al.^38^ explored the nexus between death incidence and the rate of urbanization in China using a panel threshold model. They observed that there is a single asymmetric threshold impact between population healthcare and the rate of urbanization. They also noticed that urbanization has a negative impact on the death rate when per capita GDP is above the threshold level. Wang et al.^39^ also explored the effects of urbanization on health risk factors, including population mortality and individuals visiting medical centers in China, over the period from 2004 to 2019 using nonlinear estimation techniques. They observed that an increase in urbanization causes the number of visits to medical centers to increase and the average rate of population mortality to reduce. These studies primarily addressed demographic and urbanization factors but may not comprehensively account for the influence of socio-economic factors. Gaps exist in understanding how income disparities, access to education, and price inflationary shocks may interact with urbanization to affect health outcomes.

The fourth category of studies explored the impact of air pollution, energy consumption, CO_2_ emissions, climate change, and other pollutant predictors on various health outcome indicators. While attempting to emphasize contemporaneous links between the predictors, most, if not all, have highlighted similar findings. Conceicao et al.^40^ investigated the relationships between air pollution and the child mortality rate in Sao Paulo, Brazil, from 1994 to 1997. Using daily observations for respiratory diseases-related child mortality and pollutant concentration (sulfur dioxide, carbon monoxide, PM_10_, and O_3_), they employed a generalized additive model. The study revealed that sulfur dioxide was responsible for 15% of deaths, while carbon monoxide and PM_10_ caused over 13%, and 7%, respectively. Coyle et al.^41^ assessed the effects of air pollution on mortality rates and quality-adjusted life expectancy. Using Monte Carlo simulation and data from the American Cancer Society, the study indicated that a reduction in air pollution significantly increased average life expectancy, with a gain influenced by individuals’ educational status—higher for males than females. Yin et al.^42^ focused on the public health consequences of air pollution in 33 Chinese provinces from 1990 to 2017. Using the Global Burden of Diseases, Injuries, and Risk Factors Study 2017, the authors estimated the exposure of public health to PM_2.5_. The mean PM_2.5_ exposure in China decreased by 9% from 1990 2017, but the study found that 1.24 million people still died due to air pollution. Anwar et al.^43^ explored the effects of PM_2.5_ on child mortality in 16 Asian countries. Employing a two-stage least squares method, they linked variations in PM_2.5_ to overall economic growth, finding that a one-unit increase in PM_2.5_ led to an almost 14.5% increase in the child mortality rate. Tsai et al.^44^ examined the effects of ambient PM_2.5_ on life expectancy in Taiwan from 2000 to 2020. They observed a positive link between a reduction in PM_2.5_ and higher life expectancy. Accounting for socioeconomic indicators, their results suggested that every unit decrease in PM_2.5_ increased life expectancy by an average of four months. Erdoğan et al.^45^ investigated the effects of carbon emissions on health indicators in Turkey from 1971 to 2016, finding that an increase in carbon emissions correlated with decreased life expectancy at birth and an increased infant mortality rate. For a comprehensive review, refer to Chersich et al.^46^. These studies have laid a valuable foundation for intricate analyses of the relationship between pollutant predictors and health outcomes. However, the only evident gap is their neglect of the role of exogenous variables, particularly institutional quality.

With respect to the fifth category of empirical studies, the existing literature is still evolving to recognize external shocks as independent causal agents that affect health outcomes, though some studies have attempted to customarily measure the impact of external shocks on the subject. Hopkins^47^ explored the effects of economic uncertainty, proxied by declines in growth, on health outcomes. They demonstrated that although the decline in growth rate has been ongoing since 1997, it exerted a significant impact on mortality rates in Indonesia and Thailand and little in Malaysia. Moreover, Astell-Burt and Feng^48^ examined the impact of economic shocks on health through secondary conduits—the unemployment rate, which substantially rose during the economic recession in 2008. The authors found that an increase in the unemployment rate caused a significant reduction in the prevalence of poor health status among people and enhanced their exposure to various diseases. Likewise, Tiwari and Zaman^49^ explored the effects of the food price shock on the prevalence of undernourishment during 2008 and 2009. They found that the spike in global food prices increased the general rate of undernourishment by 6.8% (63 million) people worldwide. Bao et al.^50^ explored the effects of fluctuating house rents and inflationary episodes on public health outcomes (life expectancy at birth and infant mortality rate) from 1996 to 2019 in some selected developed economies. Their findings revealed that fluctuating rental prices significantly increase the infant mortality rate while simultaneously enhancing life expectancy. In a most recent study, Kawachi et al.^51^ examined the statistical link between mortality rates attributed to cardiovascular disease and economic uncertainty in England and Wales from 2001 to 2019. The authors controlled for the effects of several macroeconomic variables and found that economic uncertainty has a significant and strong link with the number of deaths that are attributed to cardiovascular diseases, implying that a more volatile economic condition causes the rate of mortality to increase. These studies have built a rational foundation for augmenting external economic shocks in models analyzing the impact of pollutant predictors on health outcomes; however, further studies are required to complement a broader scenario analysis of the external shocks with their countering indicators, such as enhanced institutional arrangements.

The final category in this inquiry explores the impact of institutional quality on health outcomes. Despite the recognition that institutional quality has spillover effects on key determinants of health outcomes through secondary variables, the existing literature has limited studies on this matter. In Sub-Saharan African countries, Makuta and O’Hare^52^ investigated the effects of governance quality on the relationship between public health spending and health outcomes (mortality rate of under-five and life expectancy at birth) from 1996 to 2011. They observed that increased public health spending leads to improvements in overall health outcomes, and enhancing governance quality further improves the relationship between health outcomes and public health spending across Sub-Saharan Africa. Dhrifi^53^ assessed the role of institutional quality on the impact of government health expenditures on infant health outcomes using a two-step Sys-GMM approach in developing and developed countries from 1995 to 2015. Findings indicated that public health expenditure significantly reduces the rate of infant mortality in high-income economies, while it remains insignificant in lower- and upper-middle-income countries. The study also demonstrated that institutional quality plays a significant role in improving the relationship between infant mortality and public health expenditures. De Luca et al.^54^ examined how institutional quality adjusts the rationality of healthcare provisions in selected hospitals in Italy. Focusing on the provision of healthcare in cesarean sections, they found that institutional quality effectively decreases the rate of cesarean delivery in hospitals by 0.10%. Ibukun^55^ used a two-stage least squares method and a dataset for a panel of African countries from 2000 to 2018 to test the impact of institutional quality on three health outcome indicators. The study noted that health expenditures significantly affect health outcomes, suggesting a negative link between health expenditures and mortality rates but a positive association with life expectancy at birth moderated by the quality of good governance. Sharma et al.^56^ explored the impact of economic institutional quality on health outcomes in EU member countries from 2000 to 2018. Their findings indicated that improving the quality of economic institutions leads to a more positive health effect. Specifically, regulatory quality, the efficacy of legal systems, and the stability of major macroeconomic variables were identified as essential factors for improving health outcomes. However, while these studies have scrutinized the role of institutional quality in governing the predictors impacting health outcomes, the moderating effects of institutional quality to highlight cross-sectoral arrangements in improving contemporary health outcomes are missing.

The thorough examination of the existing literature exposes a notable gap: despite a considerable number of studies evaluating the influence of various socioeconomic, demographic, environmental, and institutional factors on health outcomes, there remain discernable shortcomings, notably the absence of exogenous predictors’ impact and their corresponding counteractive elements. This identified gap creates a significant opportunity for the present study to address and contribute to the contemporary body of knowledge. Furthermore, while exceptions can be found in the works of Beyene^57^ in Sub-Saharan Africa and Banerjee et al.^58^ in the United States, our extensive review reveals that the effects of air pollution on child mortality rate and life expectancy have not been extensively studied, specifically, in the top 20 polluting countries. Therefore, to comprehensively address the identified gaps, the study articulates four key measurable hypotheses: H_1_: Food insecurity, socioeconomic, demographic, and environmental factors have significant impacts on health outcomes. H_2_: The impact of inflationary shocks and economic uncertainty shocks are significantly influential on health outcome indicators. H_3_: Institutional quality is substantive in improving health outcomes as a direct causal agent. H_4_: Institutional quality plays an influential moderating role to improve the relationship between food insecurity factors and health outcomes. H_5_: Institutional quality arrangements are supportive in mitigating the negative impacts of external shocks on health outcomes.

## Methodology

### Conceptual framework

**Figure 4:**
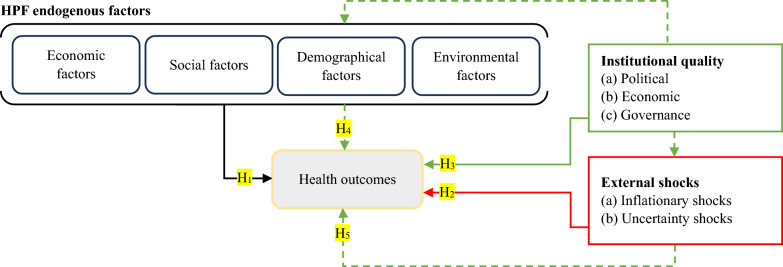
Conceptual framework; HPF extended. Note: H_1_ to H_5_ denote the hypotheses, solid lines and dashed lines indicate direct and moderating effects, respectively. Source: Authors’ creation.

The health production function (HPF) under Pole and Grossman’s^59^ theoretical model is the first empirical assumption in the existing literature. However, Auster et al.^60^ were the first authors to employ the Cobb-Duglas production function^61^ in a health-specific context. The HPF postulates that health is an asset born along with people and impairs over time, thus following a cyclical trend. The initial idea of the HPF was based on the treatment of health as capital goods in which further investments and curation enhance its productive lifespan (say, life expectancy), while a useful lifespan of a man ends with either death or complete disability^62^. The initial HPF model has evolved as different health-impairing (diseases) and health-advancing factors (curation) came into play^63^. The theoretical framework therefore states that health outcome is a function of various inputs, including the amount of food intake, level of personal disposable income, state of personal and social awareness, personal endowment (genetics), and social environment^64^. Based on this background and to test the developed hypotheses, we conceptualize our study and extend the HPF with an exogenous variable (institutional quality) and two major external shock predictors, as displayed in Fig. 4. We form a new argument that health outcomes are significantly influenced by the direct and indirect intervention of externalities and the quality of institutions in an economy.

With reference to the conceptual framework (Fig. 4), the study extends the HPF of Pole and Grossman’s^59^ with a special focus on the effects of exogenous variables and external shocks on the subject and proposes the following baseline equation:

1 $$\begin{gathered} Y_{it} = \psi_{i} + \theta \kappa_{it} + \vartheta_{1i} PM_{2.5} ,_{it} + \vartheta_{2i} RGDP_{it} + \vartheta_{3i} SER_{it} + \vartheta_{4i} PGR_{it} \, + \vartheta_{5i} URB_{it} \hfill \\ \,\,\,\,\,\,\,\,\,\,\,\,\,\,\,\,\, + \vartheta_{6i} PEC_{it} + \vartheta_{7i} PGHG_{it} + \vartheta_{8i} INS_{it} \, + \vartheta_{9i} EVS_{it} \, + \vartheta_{10i} AIQ_{it} + u_{it} \hfill \\ \end{gathered}$$

where ${Y}_{it}$ refers to the dependent variables, including life expectancy (LEP) and infant mortality rate (*IMR*), ${\psi }_{i}$ is the intercept, ${\theta }_{i}$ refers to the coefficient of the unobserved fixed effects of countries ${\kappa }_{it}$, ${\vartheta }_{1i}$ to ${\vartheta }_{10i}$ present the long-run coefficients of the explanatory variables, including particulate matter (PM_2.5_), per capita GDP (PCGDP), school enrollment ratio (SER), population growth rate (PGR), urbanization (URB), per capita energy consumption (PEC), per capita greenhouse gas emissions (PGHG), inflationary shocks (INS), economic volatility shock (EVS), aggregate institutional quality index (AIQ), and ${u}_{it}$ is the error term of the model. Equation (1) captures the long-run effects of the explanatory variables on the subject; nevertheless, ${AIQ}_{it}$ has only been augmented as an aggregated variable that captures the overall effects of institutional quality on the dependent variables. We further motivate the study and extend Eq. (1) with dimensional effects of ${AIQ}_{it}$ as described in Table 1 using the following equation:

2 $$\begin{gathered} Y_{it} = \psi_{i} + \theta \kappa_{it} + \vartheta_{1i} PM_{2.5} ,_{it} + \vartheta_{2i} RGDP_{it} + \vartheta_{3i} SER_{it} + \vartheta_{4i} PGR_{it} \, + \vartheta_{5i} URB_{it} + \vartheta_{6i} PEC_{it} \hfill \\ \,\,\,\,\,\,\,\,\,\,\,\,\,\,\,\,\, + \vartheta_{7i} PGHG_{it} + \vartheta_{8i} INS_{it} \, + \vartheta_{9i} EVS_{it} + \vartheta_{10i} PII_{it} + \vartheta_{11i} GII_{it} + \vartheta_{12i} EII_{it} + u_{it} \hfill \\ \end{gathered}$$

**Table 1:** Preliminary computation of aggregate and dimensional indices.

Classification	WGI indicators	Symbol	Obs	Mean	Std. Dev	Coefficient of variation	Assigned weight $({W}_{it})$	Normalized values $(N)$
PII	Voice and accountability	*VA*	440	0.265	0.170	0.640	0.396	0.440
	Political stability	*PS*	435	0.280	0.273	0.975	0.582	0.646
GII	Government effectiveness	*GE*	436	0.350	0.245	0.701	0.503	0.558
	Regulatory quality	*RQ*	436	0.347	0.240	0.694	0.481	0.535
EII	Rule of law	*RL*	440	0.322	0.241	0.747	0.469	0.521
	Control of corruption	*CC*	440	0.300	0.254	0.846	0.531	0.590

Obs. = Observations, Std. Dev. = Standard deviation.

Having all the variables and vectors explained earlier, ${\vartheta }_{10i}$ to ${\vartheta }_{12i}$ refer to the long-run impact of political, governance, and economic dimensions of institutional quality on *LEP* and *IMR*. Equation (2) helps us identify which dimension of institutional quality has higher explanatory power and what specific policy implications can be extracted. Furthermore, to comprehend the analysis and support specific policy implications, we delve into the moderating role of the exogenous variable (say, *AIQ*) on the subject and specify the following long-run panel equations:

3 $$\begin{gathered} Y_{it} = \psi_{i} + \theta \kappa_{it} + \vartheta_{1i} PM_{2.5} ,_{it} + \vartheta_{2i} RGDP_{it} + \vartheta_{3i} SER_{it} + \vartheta_{4i} PGR_{it} + \vartheta_{5i} URB_{it} \hfill \\ \,\,\,\,\,\,\,\,\,\,\,\,\,\,\,\,\, + \vartheta_{6i} PEC_{it} + \vartheta_{7i} PGHG_{it} + \vartheta_{8i} INS_{it} + \vartheta_{9i} EVS_{it} + \vartheta_{10i} \left( {AIQ_{it} \times MVs_{it} } \right) + u_{it} \hfill \\ \end{gathered}$$

where all other variables and vectors are defined before and ${\vartheta }_{10i}$ is the long-run coefficient of the $\left({AIQ}_{it}\times {MVs}_{it}\right)$, which is the interaction term of the AIQ with other explanatory variables denoted by ${MVs}_{it}$.

### Data and variables

Based on the availability of data, the present inquiry uses datasets containing annual observations over the period from 2000 to 2021. This period offers the required data for the panel of our interest (Table 2 details the sources of compilation). Across the globe, 41% of the population is exposed to the severe risk of climate change, while the remaining 59% is exposed to the moderate and light risks of climate change. Over one-third of the world’s population (2.55 billion as of 2021) lives in the top 20 most polluted countries, where, in addition to air pollution, many other risk factors threaten the normal lives of the people^65,66^. Thus, we consider these top 20 polluted countries reported by IQAIR^67^, which is classified by their annual average PM_2.5_ concentration. They include Bangladesh, Chad, Pakistan, Tajikistan, India, Oman, Kyrgyzstan, Bahrain, Iraq, Nepal, Sudan, Uzbekistan, Qatar, Afghanistan, the United Arab Emirates, Montenegro, Indonesia, Nigeria, Armenia, and Mongolia. Furthermore, based on our conceptual framework, we selected the required variables that are consistent with prior literature. The variables are described as follows:

**Table 2:** Variables’ definition, symbols, and sources.

Variables’ full name	Symbols	Unit of measurement	Sources of compilation
Life expectancy	LEP	At birth	WDI^100^
Infant mortality rate	IMR	Per 1,000 live infants	WDI^100^
Particulate matter 2.5	PM_2.5_	Annual average	WU-StL^101^
Urbanization	URB	Percentage of total population	WDI^100^
Population growth	PGR	Percentage of total population	WDI^100^
Per capita gross domestic product	GDP	Constant 2015 US dollar	WDI^100^
GDP deflator	GDP-DEF	Annual percentage	WDI^100^
Energy consumption	PEC	Kilowatt-hour per capita	WDI^100^
Greenhouse gas emissions	PGHG	Tons of carbon dioxide-equivalents per capita	OWD^102^
School enrollment rate	SER	Gross percentage of the total enrollment	WDI^100^
Consumer price index	INS	Annual rate	WDI^100^
Vice and accountability	VC	Percentile	WGI^96^
Government effectiveness	GE	Percentile	WGI^96^
Rule of law	RL	Percentile	WGI^96^
Political stability	PS	Percentile	WGI^96^
Regulatory quality	RQ	Percentile	WGI^96^

WDI: World Development Indicators, WU-StL: Washington University in St. Louis, OWD: Our World in Data, WGI Worldwide Governance Indicators.

### Health outcome variables

The study employs two key indicators, such as life expectancy (*LEP*) at birth and infant mortality rates (*IMR*) per 1000 live infants. Based on prior literature, *LEP* and *IMR* are the best-fit proxies for health outcomes, *i.e.*, dependent variables. *LEP* is used as a key indicator to measure population health in a broader context^68^, and *IMR* is employed to solely measure mortality at a narrower, say, specific range of population age^69,70^.

### Explanatory variables

Our key explanatory variable is the ambient air pollution, which is measured by the PM_2.5_ concentration expressed as an annual average. Prior literature shows that exposure to PM_2.5_ is associated with respiratory and cardiovascular problems. Exposure to short- and long-term PM_2.5_ concentration increases the risk of premature death, diabetes, neurodevelopment, and cognitive function diseases^10,22,71–73^. The study also controls the effects of key socioeconomic indicators. Following Jakovljevic et al.^26^ and Raghupathi and Raghupathi ^34^, the study employs real per capita GDP (*RGDP*), expressed in constant 2015 US dollars, to measure the real economic power of individuals covering health spending. *RGDP* was derived as the nominal per capita GDP over the annual GDP deflator. Consistent with studies by Luy et al.^74^ and Malamud et al.^75^, the study controls for the effects of education as a social factor, measured by the gross school enrollment rate (*SER*), on the subject. *SER* is expressed as a gross percentage of the total enrollment in the countries under review. Further, the study employs two variables, i.e., population growth rate (*PGR*) and urbanization (*URB*, % of total population), to capture the effects of demographic factors on health outcome indicators. Increasing population rates and urbanization contribute to higher traffic congestion and energy consumption, which positively contribute to higher air pollution, thus consequently impeding population health^76^. Consistent with recent empirical literature^45,77–79^, the study controls for the effects of per capita energy consumption (*PEC*) and per capita greenhouse gases emissions (*PGHG*) on the dependent variables. The selection of the explanatory variables has been carefully conducted and are referenced to the basic HPF framework.

### Exogenous variables

**Figure 5:**
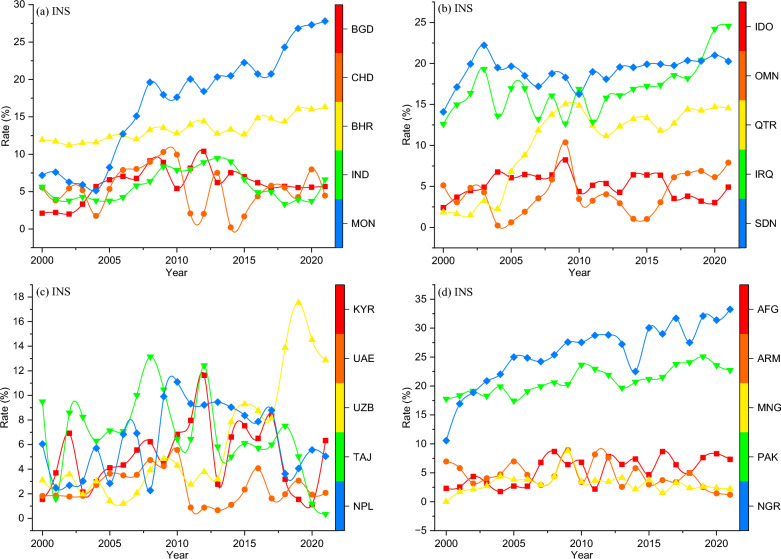
Inflationary shocks (INS) plot. Notes: BGD: Bangladesh, IDO: Indonesia, OMN: Oman, CHD: Chad, AFG: Afghanistan, ARM: Armenia, KYR: Kyrgyzstan, UAE: United Arab Emirates, UZB: Uzbekistan, MNG: Montenegro, QTR: Qatar, BHR: Bahrain, TAJ: Tajikistan, NPL: Nepal, IND: India, PAK: Pakistan, IRQ: Iraq, SDN: Sudan, NGR: Nigeria, MON: Mongolia. Source: authors’ computations.

**Figure 6:**
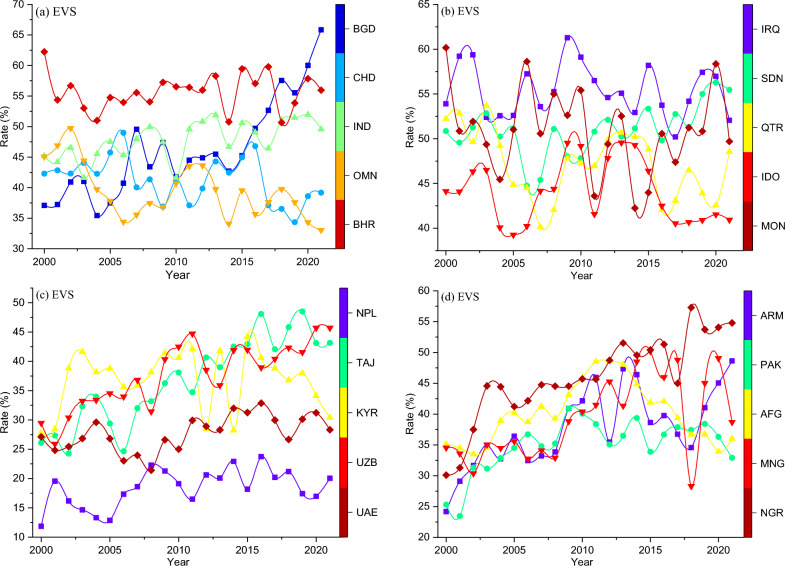
Economic volatility shocks (EVS) plot. Notes: BGD: Bangladesh, IDO: Indonesia, OMN: Oman, CHD: Chad, AFG: Afghanistan, ARM: Armenia, KYR: Kyrgyzstan, UAE: United Arab Emirates, UZB: Uzbekistan, MNG: Montenegro, QTR: Qatar, BHR: Bahrain, TAJ: Tajikistan, NPL: Nepal, IND: India, PAK: Pakistan, IRQ: Iraq, SDN: Sudan, NGR: Nigeria, MON: Mongolia. Source: authors’ computations.

After reviewing recent empirical literature (Sect. 2), it became apparent that previous studies have overlooked the impact of crucial exogenous variables on the dynamics of air pollution and its subsequent effects on health outcomes. These variables, whether operating directly or through their association with endogenous factors, have been neglected. Given the recent surges in global inflation, compounded by significant economic uncertainties stemming from global financial crises^80^, pandemics^81,82^, civil conflicts^83,84^, persistent cost-push and demand-pull inflation trends^85–87^, and the dynamic nature of political-economic factors such as wealth oil, labor market fluctuations, and material price volatility, as well as overarching governance interventions at both general and sector-specific levels within our study panel, it is imperative to comprehensively capture their effects on contemporary health outcomes. Thus, we introduce three pivotal exogenous variables, namely, inflationary shocks (*INS*), economic volatility shocks (*EVS*), and an expected countermeasure—a measure of institutional quality index. To construct the *INS* and *EVS*, we employ annual datapoints of the consumer price index inflation rate and annual GDP growth rate, respectively. The study employs the standard generalized autoregressive conditional heteroskedasticity (GARCH) model as follows:

4 $$h_{\omega ,t} = \psi + \eta \vartheta_{\omega ,t - 1}^{2} + \gamma \varepsilon_{\omega ,t - 1}^{{}}$$

where $h$ presents the conditional volatility of our variables $\omega$, namely, the consumer price index inflation rate and GDP growth, $\psi$ is the average shock, $\eta$ refers to the ARCH (autoregressive conditional heteroskedasticity) effects parameter, and $\vartheta$ is the GARCH parameter of $\omega$, $\gamma$ is the conditional error variance of $\omega$, say, the annual consumer price index inflation rate and GDP growth rate, and $t$ refers to the time period from 2000 to 2020. Due to its accuracy and comprehensiveness, similar approach has been adopted to generate volatility shocks by prior studies^88–90^. Figures 5 and 6 show the cross-unit time-horizon plot of the generated *INS* and *EVS*, respectively.

It is crucial to note that poor institutional quality alone can significantly contribute to the cause of air pollution causes^91,92^. Inadequate institutional governance, limited political capacity, and inefficient regulatory frameworks fail to adequately regulate key polluters, thereby heightening geographical vulnerability to air pollution crises. Therefore, diverging from prior studies, we incorporate the impact of institutional quality on health outcome indicators by constructing an aggregate institutional quality index (*AIQ*) alongside dimensional indices such as political institutional index (*PII*), economic institutional index (*EII*), and governance institutional index (*GII*). This approach allows us to explore the interplay between air pollution and health outcome indicators in the presence of both endogenous and external shock factors, considering contemporary institutional performance. To achieve this, the study adopts the technique proposed by Sarma^93^ for constructing the aforementioned institutional indices. This method stands out for several reasons. Firstly, it enables the estimation of distance-based vectors rather than merely allocating weights to variables^94^. Secondly, it permits the setting of values for lower and upper limit vectors to mitigate the bias caused by overlying integers in the constructed index^95^. Thirdly, it yields a ratio-outcome variable and adjusts for any significant outliers. The process of index construction follows a straightforward three-step procedure, as outlined below:

***Step-I***: The study employs the complete suite of six governance indicators from the Worldwide Governance Indicators (WGI), as developed by Kaufmann and Kraay^96^. These indicators encompass voice and accountability, the rule of law, government effectiveness, political stability, control of corruption, and regulatory quality, each presented on a percentile scale ranging from 0 (indicative of imperfection) to 1 (indicative of perfection). The adoption of WGI indicators as governance measures enjoys widespread international acceptance and has been extensively used in scholarly works^97,98^. For *AIQ*, all indicators are augmented, whereas for *PII*, *EII*, and *GII*, we classify the indicators based on their inherent nature. Table 1 (column 1) illustrates the dimensional classification of these indicators. Subsequently, using the coefficient of variations (${CV}_{it}$), we allocate appropriate weights (${W}_{it}$) to each indicator using the equation ${W}_{it}={CV}_{it}/{\sum }_{i=1}^{N}{CV}_{it}$ (as shown in Table 1, column 8), followed by normalization of values as follows:

5 $$N_{it} = W_{it} \sum\limits_{i = 1}^{N} {\left( {\frac{{A_{it} - LL_{it} }}{{UL_{it} - LL_{it} }}} \right)}$$

Here, $N$ refers to the normalized values (as displayed in Table 1, column 8), where ${A}_{it}$ represents the actual values of the indicators. ${LL}_{it}$ signifies the lower limit (0) while ${UL}_{it}$ denotes the upper limit (0.90, say, 90^th^ percentile rank) of the indicators. $N$ indicates the level of governance quality achieved by the nations across each WGI indicator within our panel. A higher *N* value corresponds to a higher achievement in governance quality^99^.

***Step-II***: Using the estimated $N$ and ${W}_{it}$, the study continues to compute the Euclidian distance points $(R$) of the variables achieved from point zero (worst-case scenario) to the ideal point (best-case scenario) and its inversed values ($Q$) using the following equation:

6 $$R_{it} = \frac{{\sqrt {\sum\nolimits_{i = 1}^{n} {N_{it}^{2} } } }}{{\sqrt {\sum\nolimits_{i = 1}^{n} {W_{it}^{2} } } }},\,\,\,\,Q_{it} = 1 - \frac{{\sqrt {\sum\nolimits_{i = 1}^{n} {\left( {W_{it}^{{}} - N_{it} } \right)^{2} } } }}{{\sqrt {\sum\nolimits_{i = 1}^{n} {W_{it}^{2} } } }}$$

where $R$ and $Q$ refer to Euclidian normalized and inversed normalized values of the indicators, $n$ is the number of observations in country $i$ at time $t$, and other vectors are as explained before^93^.

**Figure 7:**
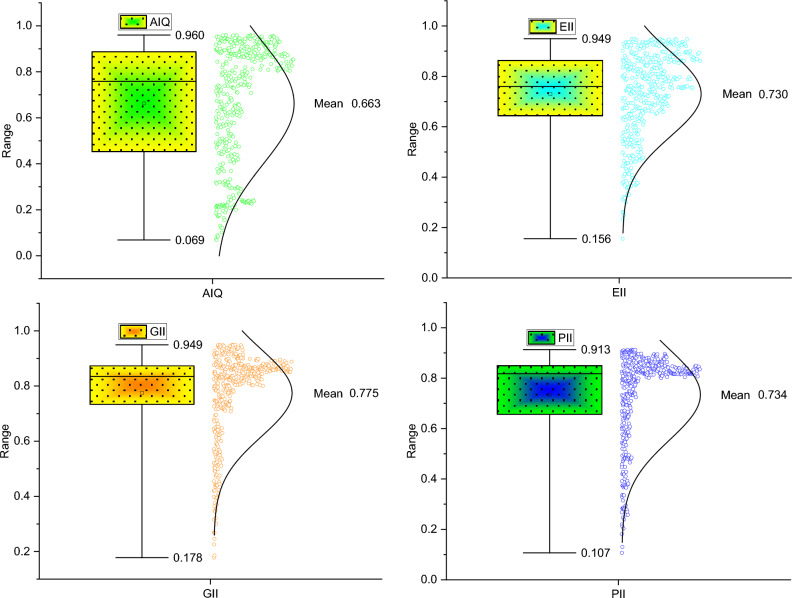
Box plot of *AIQ, EII, GII, and PII*. Source: Authors’ estimations.

***Step-III***: Having the $R$ and $Q$ computed, the study constructs the *AIQ*, *PII*, *GII*, and *EII* as follows:

7 $$AIQ_{it} = \frac{1}{2}\left( {R_{it} + Q_{it} } \right)$$

where all vectors and variables are as described earlier, *AIQ*, *PII*, *GII*, and *EII* are the constructed indices of our interest and are expressed as ratios spanning between 0 (lower) and 1 (higher). Figure 7 displays the box plot of the constructed indices.

### Estimation approach

In estimating Eqs. 1–3, the selection of appropriate panel econometric techniques holds paramount importance. While panel data analysis offers numerous advantages and a diverse array of econometric techniques such as pooled ordinary least square (POLS), fixed effects (FE), random effects (RE), panel autoregressive distributed lags (PARDL), panel quantile regressions, cross-sectionally augmented ARDL, and panel vector autoregressive (PVAR), it is essential to recognize and address their inherent limitations, particularly in capturing heterogeneity, addressing endogeneity, cross-sectional dependence, and managing extreme multicollinearity. Failure to consider these factors when choosing an estimation technique can potentially result in significant empirical challenges. Therefore, we initially conduct crucial preliminary tests to ascertain the trends and characteristics of the employed panel data. The variance inflation factor has been estimated using the pooled ordinary least squares (OLS) method to evaluate the multicollinearity among the variables. Furthermore, the autocorrelation model proposed by Drukker^103^, the heterogeneity model developed by Smith and Hsiao^104^, and the cross-sectional dependence method proposed by Pesaran^105^ are performed to verify the panel properties of the variables. Here and in subsequent regressions, Eqs. (1–3) are referred to as model I when the dependent variable is life expectancy (LEP) and model II when the dependent variable is infant mortality rate (IMR). The results of the preliminary tests shown in Table 3 indicate that the variables do not suffer from multicollinearity (VIF). A VIF mean below 10 is indicative of the absence of multicollinearity among the variables in a panel model (see, for instance^106^). Additionally, while the estimated statistics for autocorrelation in both of our models fail to reach significance to reject the null of autocorrelation (Table 3, second row), it is important to note the potential modest correlation between the lagged dependent variable $${Y}_{it-1}$$ and the error-term ($${u}_{it})$$ of the model, indicated by $$\left(cov\left({Y}_{it-1}, {u}_{it}\right)\ne 0\right)$$. This underscores that the use of POLS technique would be inconsistent in addressing this issue^107^. Moreover, even if the unobserved country-specific effect $${\theta }_{i}$$ is excluded from estimation, there might still be a correlation between $${Y}_{it-1}-{\widehat{Y}}_{it-1}$$ and $${u}_{it-1}-{\widehat{u}}_{it-1}$$. As a result, the FE model is inadequate in addressing this problem. Additionally, the results (Table 3, third and fourth rows) demonstrate the significance of the estimated statistics in rejecting the null hypothesis of homogeneity and cross-sectional independence. While the later can be rectified with by using the CS-ARDL model proposed by Chudik and Pesaran^108^, the former remains unresolved.

**Table 3:** Preliminary tests.

Tests	Model I-DV: Life expectancy			Model II-DV: Infant mortality rate		
	Equation (5)	Equation (6)	Equation (7)	Equation (5)	Equation (6)	Equation (7)
Variance inflation factor (mean)	4.28	6.39	5.44	3.98	4.12	5.18
Autocorrelation test	0.988	1.190	1.016	0.649	0.904	1.327
Heterogeneity test	28.08***	40.102***	51.396***	44.122***	40.373***	36.912***
Cross-sectional dependence test	56.915***	63.147***	60.218***	59.099***	48.084***	61.576***

*** rejects the null at a 1% significant level.

Furthermore, it is important to note that POLS, FE, RE, and the CS-ARDL model do not account for potential reverse causality in the panel^90^. Additionally, they assume to estimate a system of endogenous variables while lacking the capacity to handle a mixed system of endogenous and exogenous variables. Therefore, to overcome these empirical issues and based on the results of the preliminary tests, the present study employs the GMM model of Arellano and Bond^109^. The GMM model addresses the cited econometric issues through the instrumentation process, that is, the lags of the explanatory variables are used as instruments in the model. As such, the study would also reliably explore the effects of exogenous elements of air pollution, institutional quality, macroeconomic shocks, and inflationary shocks on life expectancy and infant mortality rates. Generally, there are two methods to estimate the GMM model: difference (Diff-GMM) and system (Sys-GMM). The Diff-GMM estimators are instrumented with the lag of the explanatory variables, assuming an idiosyncratic property for ${u}_{it}$, which is not autocorrelated. It also assumes a weak exogeneity of the explanatory variables. Nonetheless, when the variables exhibit persistence, there would be a weak instrumentation problem in estimating the Diff-GMM that would cause biasedness in finite samples. To overcome this challenge, extra moment conditions are required for an equation specified in the level form of the variables. When level-equation and difference-equation are estimated simultaneously, Sys-GMM forms. Therefore, the Sys-GMM estimators are consistent and efficient both in both balanced and unbalanced panels and are robust in the presence of endogeneity and heteroskedasticity^110^. Considering these properties, the present study uses Sys-GMM model. Sys-GMM can be estimated using one-step and two-step approaches. However, both approaches are asymptotically normal, but two-step system GMM (2Sys-GMM) estimators are more accurate and have relatively smaller variance^111^. For validity of the Sys-GMM estimators, we use two approaches: First, under the null of no second-order autocorrelation of ${u}_{it}$, say AR (2), the Arellano-Bond’s^109^ test is used. Further, Sargan’s^112^ test is employed to examine the overidentification restrictions of the instrumentation. It evaluates the null hypothesis of instrumental validity using test statistics that are asymptotically distributed. Second, following prior literature^113,114^ to test the overall robustness of the estimated coefficients obtained from the Sys-GMM, we use the dynamic ordinary least squares (DOLS) and the fully modified ordinary least squares (FMOLS) methods. All estimations are performed using EViews 13, Stata-17, Appsnet, OriginPro-2023, and R-Studio software packages.

## Results and discussion

The analysis commences with the examination of panel unit root tests. Upon rejecting the null hypothesis of cross-sectional dependence (as shown in Table 3), the study employs the cross-sectionally augmented Im, Pesaran, and Shin (CIPS) panel unit root test proposed by Pesaran^115^. The CIPS test captures the true stationarity of panel variables in the presences of cross-sectional dependence. To ensure robustness, the study also utilizes the panel unit root tests developed by Im et al.^116^, known as IPS and Levin et al.^117^, known as LLC.

The results of the CIPS, IPS, and LLC methods, presented in Table 4, collectively suggest that while URB, PGR, RGDP, PEC, PGHG, and SER do not exhibit insignificance to reject the null hypothesis of non-stationarity at the level, the remaining variables demonstrate significance at the level. These results indicate a mixed integrating order for the variables, with some following I (0) and others an I (1) series. The findings suggest further exploration into the long-run association of the variables. For this purpose, the study employs the panel cointegration test proposed by Westerlund^118^, which is suitable for panels exhibiting confirmed cross-sectional dependence. The results of Westerlund’s test for both model I, with life expectancy as the dependent variable, and model II, with infant mortality rate as the dependent variable, are displayed in Table 5.

**Table 4:** Panel unit root results.

Variables	CIPS		IPS		LLC	
	I (0)	I (1)	I (0)	I (1)	I (0)	I (1)
PM_2.5_	−3.577***	−5.659***	−3.725***	−6.443***	−5.060***	−12.999***
URB	−0.113	−2.441***	−1.422	−2.770***	−0.996	−3.321***
PGR	−1.937	−2.748***	−1.163	−3.036***	−1.080	−6.911***
RGDP	−1.843	−3.459***	−0.984	−3.712***	−1.159	−6.564***
PEC	−1.260	−4.115***	−1.311	−4.622***	−1.158	−6.297***
PGHG	−1.431	−4.127***	−1.323	−4.305***	−1.095	−8.318***
SER	−1.802	−4.694***	−1.402	−3.918***	−1.200	−5.211***
INS	−2.941***	−5.183***	−6.425***	−11.114***	−9.735***	−12.985***
EVS	−3.100***	−4.557***	−2.426***	−4.869***	−4.038***	−7.974***
AIQ	−2.887***	−4.636***	−2.133**	−5.018***	−3.488***	−6.540***
PII	−3.503***	−5.196***	−3.251***	−6.474***	−3.770***	−7.337***
EII	−3.120***	−5.478***	−3.470***	−6.300***	−2.569***	−6.961***
GII	−3.273***	−5.883***	−2.999***	−6.456***	−3.620***	−7.564***

*** and ** indicate significance at 1% and 5% levels, respectively.

**Table 5:** Westerlund co-integration test.

Statistics	Model I-DV: LEP			Model II-DV: IMR		
	Value	Z-value	*p*-value	Value	Z-value	*p*-value
Gt	−12.544	−3.812***	0.003	−18.333	−4.609***	0.000
Ga	−14.053	−3.945***	0.001	−15.729	−4.275***	0.000
Pt	−11.524	−3.691***	0.005	−17.012	−4.911***	0.000
Pa	−14.822	−4.002***	0.001	−17.484	−5.030***	0.000

*** indicate significance at 1% level.

Westerlund’s test estimates four statistics: Gt and Ga for cross-unit cointegration, and Pt and Pa for cointegration across the whole panel. The findings in Table 5 demonstrate that all statistics are significant at the 1% level, rejecting the null hypothesis of no cointegration across both individual units and the whole panel. This suggests that the variables exhibit long-run relationships with both life expectancy and infant mortality rate in the reviewed panel. After verifying the stationarity properties and the cointegration among the variables, the study proceeds to estimate and present the baseline results in Tables 6, 7 and 8 using the 2Sys-GMM technique. Table 6 reports the direct effects of air pollution, endogenous factors, external shocks, and institutional quality on life expectancy at birth (Model I) and infant mortality rate (Model II). Additionally, Table 6 displays the results of the dimensional impact of institutional quality on the subject. Tables 7 and 8 report the moderating effects of institutional quality on the nexus between air pollution, endogenous factors, and external shocks. Underneath the coefficient’s estimates, the diagnostic checks of the computed models are presented in all tables.

**Table 6:** 2Sys-GMM estimate results.

Variables	Model I-DV: LEP		Model II-DV: IMR	
	Coefficients	*p*-value	Coefficients	*p*-value
PM_25_	−3.6901*** (−3.66)	0.002	2.94014*** (4.33)	0.000
URB	0.0835* (1.99)	0.067	−0.22802** (−2.16)	0.049
PGR	−0.019431*** (−5.01)	0.000	0.0167* (2.00)	0.099
RGDP	0.2120006*** (3.79)	0.000	−0.6467*** (−3.99)	0.000
PEC	−0.026011** (−2.67)	0.033	0.0405*** (4.67)	0.000
PGHG	−0.486*** (−4.10)	0.000	0.610*** (3.69)	0.001
SER	0.1745*** (4.37)	0.000	−0.318*** (−5.92)	0.000
INS	−0.703148* (−2.01)	0.081	0.25084*** (6.29)	0.000
EVS	−0.041117** (−2.26)	0.045	0.0449*** (4.46)	0.000
AIQ	0.660035*** (4.45)	0.000	−0.60291*** (−4.19)	0.000
Constant	−8.29*** (−9.16)	0.000	−4.018*** (5.57)	0.000
*AIQ dimensional effects*				
PII	0.46009*** (3.88)	0.000	−0.3811* (−2.02)	0.055
EII	0.89023*** (4.11)	0.000	−0.94025*** (−3.72)	0.001
GII	0.651047*** (3.92)	0.000	−0.7022*** (−4.36)	0.000
*Diagnostic checks*				
Observations	406		404	
Group	20		20	
Wald ${\chi }^{2}$	33,179.8***	0.000	39,238.5***	0.000
Sargan ${\chi }^{2}$	24.17	0.320	81.06	0.620
AR (1)	−9.13***	0.000	−5.45***	0.000
AR (2)	−0.516	0.478	−0.820	0.420

***, **, and * indicate significance at 1%, 5%, and 10% levels, respectively. AR: Arellano and Bond test for second-order autocorrelation. Values in parenthesis indicate z-statistics.

**Table 7:** Model I: Moderating effects of AIQ on LEP.

Variables	Moderating effect of AIQ on PM_2.5_ -LEP nexus	Moderating effects of AIQ on URB -LEP nexus	Moderating effects of AIQ on PGR -LEP nexus	Moderating effects of AIQ on RGDP -LEP nexus	Moderating effects of AIQ on PEC -LEP nexus	Moderating effects of AIQ on PGHG -LEP nexus	Moderating effects of AIQ on SER -LEP nexus	Moderating effects of AIQ on INS -LEP nexus	Moderating effects of AIQ on EVS -LEP nexus
PM_2.5_	−3.681*** (−4.03)	−3.617*** (−3.95)	−3.599*** (−4.03)	−3.701*** (−3.90)	−3.586*** (−4.00)	−3.411*** (−4.32)	−3.829*** (−3.99)	−3.504*** (−4.67)	−3.637*** (−3.85)
URB	0.072** (2.55)	0.066*** (4.81)	0.059** (2.56)	0.077* (1.96)	0.069*** (3.79)	0.072*** (4.10)	0.075** (2.68)	0.061*** (4.53)	0.072*** (3.91)
PGR	−0.016*** (−4.68)	−0.094* (−1.94)	−0.017* (−1.78)	−0.029** (−2.88)	−0.035** (−2.80)	−0.033** (−2.49)	−0.019* (−1.79)	−0.028*** (−4.01)	−0.018*** (−4.28)
RGDP	0.705*** (4.02)	0.699*** (4.15)	0.582*** (3.82)	0.710*** (4.10)	0.802*** (3.91)	0.657** (2.95)	0.811*** (4.13)	0.744*** (4.45)	0.691*** (4.17)
PEC	−0.023*** (−3.84)	−0.029*** (−4.03)	−0.015*** (−4.17)	−0.011*** (−3.87)	−0.020*** (−5.12)	−0.018*** (−3.77)	−0.021*** (−3.98)	−0.019*** (−4.02)	−0.022*** (−3.86)
PGHG	−0.447*** (−4.10)	−0.461*** (−6.45)	−0.419*** (−4.33)	−0.514*** (−5.10)	−0.532*** (−6.01)	−0.467*** (−4.36)	−0.429*** (−5.10)	−0.501*** (−3.99)	−0.451*** (−5.13)
SER	0.210*** (4.66)	0.244** (2.59)	0.194 (0.88)	0.315 (1.24)	0.222*** (3.77)	0.199*** (4.12)	0.204*** (−3.69)	0.211*** (3.81)	0.197* (1.88)
INS	−0.910*** (−5.44)	−0.835* (−1.99)	−0.901*** (−4.09)	−0.854*** (−4.22)	−0.936** (−2.69)	−0.941*** (−4.14)	−0.936** (−2.73)	−0.899*** (−4.24)	−0.945*** (−4.36)
EVS	−0.544* (−1.86)	−0.602** (−2.26)	−0.504** (−2.50)	−0.499** (−.281)	−0.511* (−1.84)	−0.617*** (−3.89)	−0.555*** (−4.18)	−0.508** (−2.91)	−0.611*** (−5.14)
*Moderating effects*									
AIQ × PM_2.5_	−1.281*** (−4.32)								
AIQ × URB		0.099*** (3.57)							
AIQ × PGR			−0.008** (−2.71)						
AIQ × RGDP				0.859*** (4.86)					
AIQ × PEC					−0.00008 (−0.44)				
AIQ × PGHG						−0.1208* (−1.88)			
AIQ × SER							0.599*** (6.33)		
AIQ × INS								−0.000012 (−0.89)	
AIQ × EVS									−0.037*** (−3.79)
Constant	−4.33*** (−9.44)	−8.02*** (−5.11)	−4.67*** (−3.95)	−5.83*** (−4.99)	−4.91*** (6.45)	−3.66*** (3.89)	−10.10*** (−6.12)	−9.32*** (−6.45)	−10.21*** (−8.09)
*Diagnostic tests*									
Observations	396	401	396	391	400	396	401	401	401
Group	20	20	20	20	20	20	20	20	20
Wald ${\chi }^{2}$	49,110.8***	33,152.7***	31,410.2***	28,591.4***	34,229.1***	30,419.3***	36,215.4***	33,140.9***	29,782.1***
Sargan ${\chi }^{2}$	67.36	90.22	101.03	81.45	80.76	92.15	73.44	82.19	70.35
AR (1)	−5.77***	−4.65***	−4.13***	−4.09***	−4.16***	−4.25***	−4.09***	−4.67***	−5.07***
AR (2)	−1.035	−0.789	−1.448	−1.369	−0.907	−1.055	−0.841	−1.615	−1.738

***, **, and * indicate significance at 1%, 5%, and 10% levels, respectively. Values in parenthesis indicate z-statistics. Source: Authors’ estimations.

**Table 8:** Model II: Moderating effects of AIQ on IMR.

Variables	Moderating effect of AIQ on PM_2.5_ -IMR nexus	Moderating effects of AIQ on URB -IMR nexus	Moderating effects of AIQ on PGR -IMR nexus	Moderating effects of AIQ on RGDP -IMR nexus	Moderating effects of AIQ on PEC -IMR nexus	Moderating effects of AIQ on PGHG -IMR nexus	Moderating effects of AIQ on SER -IMR nexus	Moderating effects of AIQ on INS -IMR nexus	Moderating effects of AIQ on EVS -IMR nexus
PM_2.5_	2.674*** (4.48)	2.922*** (4.00)	2.691*** (5.67)	2.811* (1.99)	2.339*** (−4.01)	2.816** (2.69)	2.375*** (−5.17)	2.688* (1.91)	2.449*** (4.28)
URB	−0.192*** (−3.86)	−0.227** (−2.59)	−0.1322* (−1.90)	−0.2804** (−2.22)	−0.214*** (−4.43)	−0.236*** (−3.95)	−0.211** (−2.79)	−0.249*** (−5.33)	−0.198*** (−4.76)
PGR	0.013*** (4.10)	0.017*** (−4.11)	0.021*** (−3.96)	0.016*** (−4.10)	0.025* (1.69)	0.0209** (2.66)	0.015*** (4.11)	0.032*** (3.87)	0.017*** (3.45)
RGDP	−0.591*** (−4.06)	−0.603*** (−4.91)	−0.692*** (−4.18)	−0.610*** (−5.33)	−0.599** (−4.82)	−0.627*** (−5.02)	−0.710*** (−5.33)	−0.681*** (−4.26)	−0.652*** (−5.16)
PEC	0.048*** (3.72)	0.039** (2.71)	0.061*** (4.14)	0.033** (2.56)	0.055*** (3.77)	0.045*** (4.12)	0.038** (2.59)	0.063*** (3.94)	0.045** (2.27)
PGHG	0.602*** (4.19)	0.428** (2.63)	0.415* (1.84)	0.598** (2.61)	0.610*** (4.41)	0.584*** (5.07)	0.649* (1.94)	0.488** (2.37)	0.519*** (4.22)
SER	−0.323*** (−4.03)	−0.335*** (−3.94)	−0.317*** (−3.59)	−0.321*** (−3.96)	−0.311*** (−6.02)	−0.309** (−2.51)	−0.336*** (−4.12)	−0.301* (−1.85)	−0.322** (−2.59)
INS	0.054*** (3.95)	0.062*** (3.82)	0.054*** (4.18)	0.067*** (4.53)	0.057** (2.91)	0.081*** (3.76)	0.069*** (3.61)	0.062*** (−4.00)	0.055*** (−4.11)
EVS	0.0018*** (3.88)	0.0016** (2.45)	0.0065*** (3.65)	0.0046*** (5.13)	0.0044*** (3.99)	0.0091*** (4.15)	0.0027*** (3.94)	0.0082*** (3.86)	0.0065*** (5.43)
*Moderating effects*									
AIQ × PM_2.5_	1.109*** (−3.95)								
AIQ × URB		−0.318*** (−4.33)							
AIQ × PGR			0.0011* (1.87)						
AIQ × RGDP				−0.416*** (−6.12)					
AIQ × PEC					0.0081** (2.65)				
AIQ × PGHG						0.299*** (−5.08)			
AIQ × SER							−0.484*** (−5.55)		
AIQ × INS								0.00086* (1.59)	
AIQ × EVS									0.00047* (1.64)
Constant	−9.301*** (−6.15)	−5.362*** (−9.45)	−4.077*** (−5.06)	−5.401*** (−4.99)	−5.336*** (−4.82)	−4.44*** (−6.15)	−5.38*** (9.11)	−10.88*** (−7.23)	−8.62*** (−5.20)
*Diagnostic tests*									
Observations	396	401	401	396	396	396	396	401	401
Group	20	20	20	20	20	20	20	20	20
Wald ${\chi }^{2}$	22,099.6***	28,214.9***	31,007.2***	19,867.5***	24,910.2***	29,111.1***	29,271.4***	35,019.2***	33,167.0***
Sargan ${\chi }^{2}$	23.45	38.12	69.37	91.33	44.18	62.35	69.99	101.04	85.36
AR (1)	−4.13***	−3.49***	−5.46***	−4.02***	−3.84***	−4.22***	−5.00***	−4.86***	−4.32***
AR (2)	−0.772	−0.901	−1.126	−0.582	−1.209	−1.076	−0.809	−1.019	−1.226

***, **, and * indicate significance at 1%, 5%, and 10% levels, respectively. Values in parenthesis indicate z-statistics.

The results presented in Table 6 underscore a compelling and adverse relationship between PM_25_ concentration and two crucial health indicators, life expectancy (LEP) and infant mortality rate (IMR), reaching statistically significant at the 1% level. The findings reveal that a one-unit increase in PM_25_ concentration corresponds to a noteworthy reduction in LEP by 3.6901 years and a simultaneous increase in IMR by 0.0294% (equivalent to 2.9 per 1000 infants expressed as a percentage). These findings shed light on the tangible impacts of air pollution on public health, emphasizing the increased susceptibility to conditions such as lung cancer, heart disease, and emphysema, ultimately contributing to a shortened LEP^119^. In a broader context, when juxtaposed with the global average of a 1.8-year decrease in life expectancy in 2019^120^, our findings expose a stark reality. The loss of years in life expectancy attributable to PM_2.5_ concentration is notably amplified, being two times higher in the most polluted countries, amounting to a substantial 3.69 years. This revelation underscores the urgent need for comprehensive interventions to address air quality concerns, particularly in regions grappling with elevated PM_2.5_ levels, to alleviate the considerable health burden posed by such environmental factors. Nonetheless, Apte et al.^16^ reported results strikingly similar to our findings. They identified a global average reduction in LEP by 1 year due to PM_2.5_ concentration, with a marginal uptick of 1.2 to 1.9 years in Asian and African nations in 2016. Additionally, our results align partially with Tsai et al.^44^, revealing a significant decrease in average LEP in Taiwan associated with an increase in PM_2.5_ concentration. In line with our outcomes, Juginović et al.^26^ highlighted that 44.6% of total mortality rates in Europe from 1990 to 2019 were attributed to air pollution. Comparing our results to Lelieveld et al.^121^, it becomes evident that the mortality rate in the most polluted countries within our panel, standing at 2.94 per 1000 infants, significantly surpasses the global average of 1.2 per 1000 and the East Asian countries’ average of 1.96 per 1000 individuals. Our findings corroborate those of Folinsbee^122^, Pope and Dockery^123^, Kampa and Castanas^124^, Burnett et al.^125^, Guo et al.^126^, Ghorani-Azam et al.^127^, Miller and Xu^128^, Almetwally et al.^129^, and Wang et al.^130^, reinforcing the observed adverse impact of air pollution on human health outcomes. Our findings imply the imperative for targeted public health policies and stringent environmental regulations to address the adverse effects of PM_2.5_ on LEP and IMR. Therefore, considering the critical importance of regional inventions, effective healthcare planning, comprehensive public awareness campaigns, and international cooperation are indispensable for mitigating health risks associated with air pollution.

In relation to the control variables (refer to Table 6), the findings reveal that per capita GHG emissions lead to a reduction in LEP by 0.486 years and an increase in IMR by 0.61 per 1000 infants, while holding other variables constant. The observed associated links between per capita GHG emissions and a reduction in life expectancy and an increase in infant mortality rate emphasizes the long-term health consequences of sustained exposure to elevated GHG levels in the top 20 polluted countries. It states that prolonged exposure may contribute to chronic health issues, emphasizing the imperative for sustained efforts to mitigate GHG emissions and safeguard public health in the long run. Studies by Esmaeili et al.^131^, Duodu and Mpuure^132^, Mahalik et al.^133^, Ansarinasab and Bidmal^134^, Bhutto et al.^135^, Gavurova et al.^79^, and Perera^3^ have also noticed the negative consequences of GHG emissions on both LEP and IMR across various nations. As found by Schaefer^136^ and Rice^137^, long-term exposure to GHG emissions causes a significant loss in resistance and coordination in the body. It also causes the lung cavity to change when the GHG concentration in the air is 0.85%, and the brain will perform abnormally due to high blood pressure when the GHG concentration exceeds 1.2% in the air. Moreover, our findings affirm the positive impact of urbanization (URB) on population health outcomes. A 1% increase in URB is associated with a notable improvement in LEP by 0.0853 years (approximately 1 month) and a concurrent decrease in IMR by 0.228 per 1,000 infants (0.0228%). Urbanization is linked to enhance economic and social progress, providing improved access to sanitation, healthcare services, employment, and higher food quality. These findings align with studies by Zhang et al.^138^, Jiang et al.^38^, Fan et al.^139^, Wang^140^, and Gong et al.^141^, which similarly found positive associations between urbanization and improved life expectancy and reduced infant mortality rates across diverse country panels. While urbanization’s health effects may vary across nations, our results support its overall positive impacts on health outcome indicators. While our results contradict Ahmad et al.’s^142^, negative impact of urbanization on health outcomes in South Asian countries, they align with those of Eckert and Kohler’s^143^ positive association of between life expectancy and urbanization, noting insignificant effects in their study. In the recipient panel, the findings underscore a discernible impact of population growth rate (PGR). The results reveal that PGR is associated with a reduction in LEP by 0.0194 years and a simultaneous increase in IMR by 0.00167%. This pattern can be attributed to the intricate relationship between population growth significantly influencing health outcome indictors. Notably, as population growth accelerates, it often leads to a higher ecological footprint, increased energy consumption, and reduced access to healthcare facilities due to heightened population density. These factors collectively contribute to the observed negative impact on health outcomes. Our results are consistent with prior literature. Recent studies by Azimi and Rahman^95^, Miladinov^144^, Majeed and Ozturk^145^, Boz and Ozsarı^146^, Chaabouni et al.^147^, Kibirige^148^ have independently corroborated our results. Furthermore, our findings reveal that real per capita GDP positively influences LEP but exerts a negative impact on IMR in the recipient panel. Specifically, a US$1,000 increase in real per capita GDP correlates with a 2.54 month rise in LEP and a 0.065% decrease in IMPR. This underscores the beneficial effects of higher per capita income levels, facilitating improved healthcare access, affording better food quality, and enough food intake. Nevertheless, the environmental impact of growth remains a subject of debate, as noted by Marques et al.^149^. Our findings align with prior studies by Niu and Melenberg^27^, Lange and Vollmer^150^, Luo and Xie^151^, Miladinov^144^, and Niu et al.^152^, supporting the positive association between per capita GDP and LEP. However, our results diverge from the conclusions drawn by Cutler et al.^153^ and Kaur^154^, who reported contradicted findings, suggesting a determinantal impact of per capita income on life expectancy and mortality rate, respectively. Additionally, our findings reveal a noteworthy impact of per capita energy consumption (PEC) on LEP and IMR in the recipient panel. Specifically, higher PEC is associated with a negative influence on LEP and a simultaneous positive effect on IMR. These outcomes resonate with the recognized connection between heightened per capita energy consumption and environmental degradation, posing health risks to populations^155^. Our findings align with the results of other studies; Beyene^57^, Taghizadeh-Hesary et al.^156^, and Heflin et al.^157^ similarly report adverse effects of energy consumption on health outcomes. Recognizing the broader implications of higher energy consumption on public health outcomes underscores the importance of sustainable practices to mitigate the associated risks and promote overall well-being. A viable solution could involve transitioning to renewable energy sources. As a pivotal social factor, our investigation incorporates school enrollment rate (SER) into the analysis, revealing its significant impact at a 1% level on both LEP and IMR. A 1% increase in SER corresponds to a significant increase of 2.094 months in LEP and a simultaneous decrease of 0.025% in IMR. It is linked to the fact that higher SER positively impacts population health by promoting education and health literacy. Educated individuals are more likely to adopt preventive health measures, leading to improved maternal and child health, Education also enhances economic opportunities, addressing social determinants of health and reducing risky behavior. Additionally, higher SER contributes to community awareness, fostering health equity and creating healthier societies overall. Our findings are consistent with prior studies by Ali and Ahmad^158^, Hansen and Strulik^159^, Azam et al.^160^, and Rahman and Alam^161^ have also established the substantially positive influence of SER on the overall health status of populations.

Deviating from the existing literature, this study examines the effects of exogenous variables on the subject. The results demonstrate that inflationary shock (INS) is statistically significant at a 1% level, leading to a reduction in LEP by 8.5 months (0.703148 × 12) and an increase in IMR by 0.025%. The results imply that in countries with lower per capita income, the shock effects of inflationary episodes are particularly severe, contributing to higher mortality rates and lower life expectancy. INS plays a significant role in the volatility of prices in essential sectors such as food, healthcare services, and insurance, impacting the purchasing power of a nation and consequently affecting overall population well-being^162^. Persistent exposure to inflationary shocks over the long-term leads to economic instability, suppressed of economic performance, withdrawal of major investments, limited flow of money through the economic cycle, and negative impacts on social factors and health outcomes. Furthermore, an analysis of the impact of inflationary shocks is incomplete without considering the subsequent effects of economic uncertainty on heath outcome indicators. To address this, we constructed and incorporated the economic uncertainty (EVS) into our analysis. The results in Table 6 reveal that previous-period uncertainties significantly impact health outcomes. An increase in EVS corresponds to a 0.493 (approximately 15 days) reduction in LEP and an increase in IMR by 0.0045% in the recipient panel. Both variables, INS and EVS, are supplementary, compelling individuals to cut off normal food supplementation due to higher prices, limiting access to healthcare facilities, and restoring to low-quality substances for heating. consequently, household, and ambient pollution increase and body resistance decrease, leading to a suppression of health outcome indicators. As noted by Bao et al.^50^, inflation is shown to be detrimental to health outcomes. Furthermore, our results are partially consistent with those of Hopkins^47^, McDaid et al.^163^, Glonti et al.^164^, Ruhm^165^, and Antonova et al.^166^, who demonstrate that periods of economic slowdown has negative health consequences. However, our results diverge from the conclusions drawn by Ahmad et al.^167^ and Prędkiewicz et al.^168^. These studies observe that higher inflationary periods and economic uncertainty paradoxically improve air quality by discouraging certain types of investment.

To underscore more specific macro-policy interventions, the study augmented the aggregate institutional quality index (AIQ) into the estimations presented in Table 6. The results indicate that AIQ is highly significant in directly improving health outcomes in recipient nations. Specifically, a 1% increase in AIQ leads to an improvement in LEP by 7.92 months (0.660035 × 12) and a decrease in IMR by 0.06%. The nexus between institutional quality and health capital formation is established through the control of corruption, the rule of law, political stability, and the improvement of the administration of public health services. However, these results contribute to a general understanding of policy considerations. To provide more specificity in areas of policy interventions that would facilitate swift and accurate decision-making, the study estimated the dimensional impact of institutional quality on the subject. The results reveal that economic institutional index (EII) has a relatively higher impact on health outcomes, increasing LEP by 10.68 months and decreasing IMR by 0.094%. In comparison, the governance institutional index (GII) is significant in increasing LEP by 7.8 months and decreasing IMR by 0.07%. Furthermore, the political institutional index (PII) has also been found to improve LEP by 5.5 months and reduce IMR by 0.038%. Among the three institutional quality dimensions, political governance and institutional governance are found to have a lower impact on the subject. This highlights that overall political governance and cross-sector favors in coordinative policy formulation and implementation perform relatively poorly in advancing their institutional output to improve health outcomes. However, our results are consistent with prior studies by Hadipour et al.^169^, Azimi et al.^90^, Rahman and Alam^170^, Jafari et al.^171^, Bousmah et al.^172^, Brinkerhoff and Bossert^173^, Kaini^174^, and Kirigia and Kirigia^175^ in the general context; our findings are unique and specify key policy areas where clear interventions are sought.

With all other variables held constant, as interpreted previously, we examined the moderating impact of aggregated institutional quality index (AIQ) on the relationships between the dependent variables and explanatory variables. These estimations are grounded in our initial argument that institutional quality could be effective across all sectors of an economy, generating improved health outcomes. Thus, its association with social, environmental, and demographic factors would be significant, highlighting precise policy implications. The results in Tables 7 and Table 8 underscore the substantial impact of AIQ on mitigating the adverse effects of PM_2.5_ concentration, highlighting its potential as an effective intervention. Specifically, AIQ reduces the impact on LEP from 3.681 years to 1.281 years and on IMR from 0.2674 to 0.1109%. This substantial reduction, amounting to 65.2% for LEP and 0.156% for IMR, emphasizes the importance of robust institutional quality in enhancing population health. Our findings align with the work of Rehmat et al.^176^ and Rahman and Alam^170^, albeit utilizing different proxies. They, too, indicate the positive influence of institutional quality on health outcomes, particularly life expectancy. This consistency in results across studies reinforces the robustness of the effective moderating role of institutional quality on the relationship air pollution and health outcomes. Turning to other control variables, our findings reveal that AIQ significantly diminishes the impact of per capita GHG emissions on health outcomes. The reduction in the effects of GHG emissions on LEP from 0.617 years (7 months) to 0.1208 (1.5 months) and on IMR from 0.061 to 0.0299% reveals the broader implication of institutional quality in effectively moderating the impact of environmental degradation on public health. This aligns with Prior studies by Ibrahim and Law^177^, Salman et al.^178^, Khan and Rana^179^, Haldar and Sethi^180^, Yuan et al.^181^, and Nguyen et al.^8^, indicating consistent findings across different geographical contexts. Overall, our findings emphasize the integral role of institutional quality in shaping health outcomes. The findings not only corroborate existing literature but also contribute novel insights, especially in understanding how AIQ moderates the impact of environmental factors on health. These nuanced insights can guide policymakers in formulating targeted interventions, ultimately fostering improved health outcomes in the top twenty polluted countries.

Furthermore, the findings reveal that AIQ is substantive to slightly improve the positive impact of URB from 0.066 years to 0.099 years (approximately 33%) and IMR from 0.0228 to 0.0318%. Urbanization is a major factor that principally stimulates urban density^182^, which affects environmental quality and influences health outcomes. Institutional quality improves the pattern of human behavior in the process of urbanization and enhances the positive effects of urbanization on the population’s health outcomes. Recent studies such as Sun et al.^183^, Abaidoo and Agyapong^89^, and Azam et al.^184^ partially support our findings. The results also indicate that AIQ effectively moderates to reduce the negative impact of PGR from 0.019431 to 0.008 years on life expectancy and infant mortality rate from 0.0016 to 0.00011%. Further, AIQ is found to improve the positive impact of per capita real GDP on life expectancy from 0.212 to 0.859 years and reduce the infant mortality rates from 0.064 to 0.041% across the panel. This is linked to the fact that control of corruption, higher government effectiveness, efficiency, and quality of regulations, the rule of law, and voice and accountability improve the efficiency of economic resources, which, in turn, positively influences the enhancement and improvement of health outcomes. Our findings are consistent with those of Nawaz et al.^185^, Nirola and Sahu^186^, and Wandeda et al.^187^, who have also noticed similar results in their studies considering various economies. Moreover, the findings show that AIQ is effective in offsetting the negative effects of per capita energy consumption on life expectancy. However, the interaction term between AIQ and PEC indicates that the effects of per capita energy consumption on infant mortality rates decrease from 0.0045 to 0.0018%. Furthermore, it has been discovered that AIQ has effective moderating impacts on the relationship between life expectancy and school enrollment rate. It increases life expectancy from 0.1745 to 0.599 years while improving the effect of school enrollment rates on lowering infant mortality rates from 0.0318 to 0.0484%. These outcomes partially support the findings of Opeloyeru et al.^188^, Tadadjeu et al.^189^, Iddrisu et al.^190^, Alimi and Ajide^191^, Dhrifi^53^, and Asgher et al.^192^, who also found the direct and spillover effects of institutional quality on health outcome indicators across different economic contexts. Interestingly, AIQ has been found to be effective in reducing the negative impact of inflationary shocks and economic volatility shocks on both life expectancy and infant mortality rates in the panel under review. However, the existing literature is substantively scarce in discussing the spillover of the exogenous predictors on health outcomes; Asamoah et al.^193^ and Azimi and Rahman^90^ were found to be exceptional in supporting the idea that institutional quality has an effective moderating role in offsetting the macroeconomic uncertainty shocks through the enhancement of the quality of institutional performance across all sectors of an economy.

### Robustness tests

In assessing the validity of the results derived from the 2Sys-GMM model, the estimates underwent testing against various spuriousness issues. First, diagnostic checks of the estimates have been reported underneath Tables 6, 7 and 8. All estimated results are spurious-free. For example, the chi-squared statistics of the Arellano-Bond test (say, AR (2)) are insignificant and do not reject the null of no second-order autocorrelation. This implies that the explanatory variables are not correlated with ${u}_{it}$. Furthermore, the chi-squared statistics of the Sargan test are significant to verify the instrumental validity of the estimates across all results. This adds a layer of confidence to the reliability of the estimates. Second, to ensure the robustness of the outputs obtained from the GMM model, we estimated both dynamic ordinary least squares (DOLS) and fully modified ordinary least squares (FMOLS) models and presented their results in Table 9. Although the size of the coefficients obtained varies from those of the GMM estimates, similar signs and significance are achieved, implying that the models are statistically stable.

**Table 9:** DOLS and FOMLS test results.

Variables	Model I		Model II	
	DOLS	FMOLS	DOLS	FMOLS
PM_25_	−3.914*** (−4.15)	−3.845*** (−6.13)	2.671*** (5.88)	2.379*** (4.91)
URB	0.129*** (7.36)	0.115* (1.59)	−0.0922*** (−6.19)	−0.103** (−2.18)
PGR	−0.145*** (−8.22)	−0.202** (−2.67)	0.111*** (5.74)	0.198*** (−4.22)
RGDP	1.005*** (5.11)	0.906*** (5.68)	−0.412* (−1.65)	−0.710*** (−4.22)
PEC	−0.107*** (−6.31)	−0.219* (−1.61)	0.103** (2.28)	0.162*** (−3.99)
PGHG	−0.705*** (−4.37)	−0.439*** (−4.66)	0.244*** (5.33)	0.811*** (4.45)
SER	0.386* (1.92)	0.510*** (3.89)	−0.227*** (−7.01)	−0.231*** (−4.11)
INS	−0.399*** (−6.49)	−0.169*** (−4.05)	0.154*** (4.68)	0.207*** (−3.77)
EVS	−0.0099*** (−6.14)	−0.00061** (−2.93)	0.0017*** (5.11)	0.00082** (2.28)
AIQ	0.403*** (3.97)	0.519*** (6.44)	−0.802*** (−9.43)	−0.629*** (−4.95)
Constant	−4.815*** (−6.12)	−9.111*** (−7.10)	−3.502*** (−8.11)	−6.405*** (−9.29)
*Diagnostic checks*				
R−squared test	0.671	0.514	0.644	0.710
Residuals’ normality test	1.044	0.908	0.829	1.537

***, **, and * indicate significance at 1%, 5%, and 10% levels, respectively. Source: Authors’ estimations.

### Summary of results

**Figure 8:**
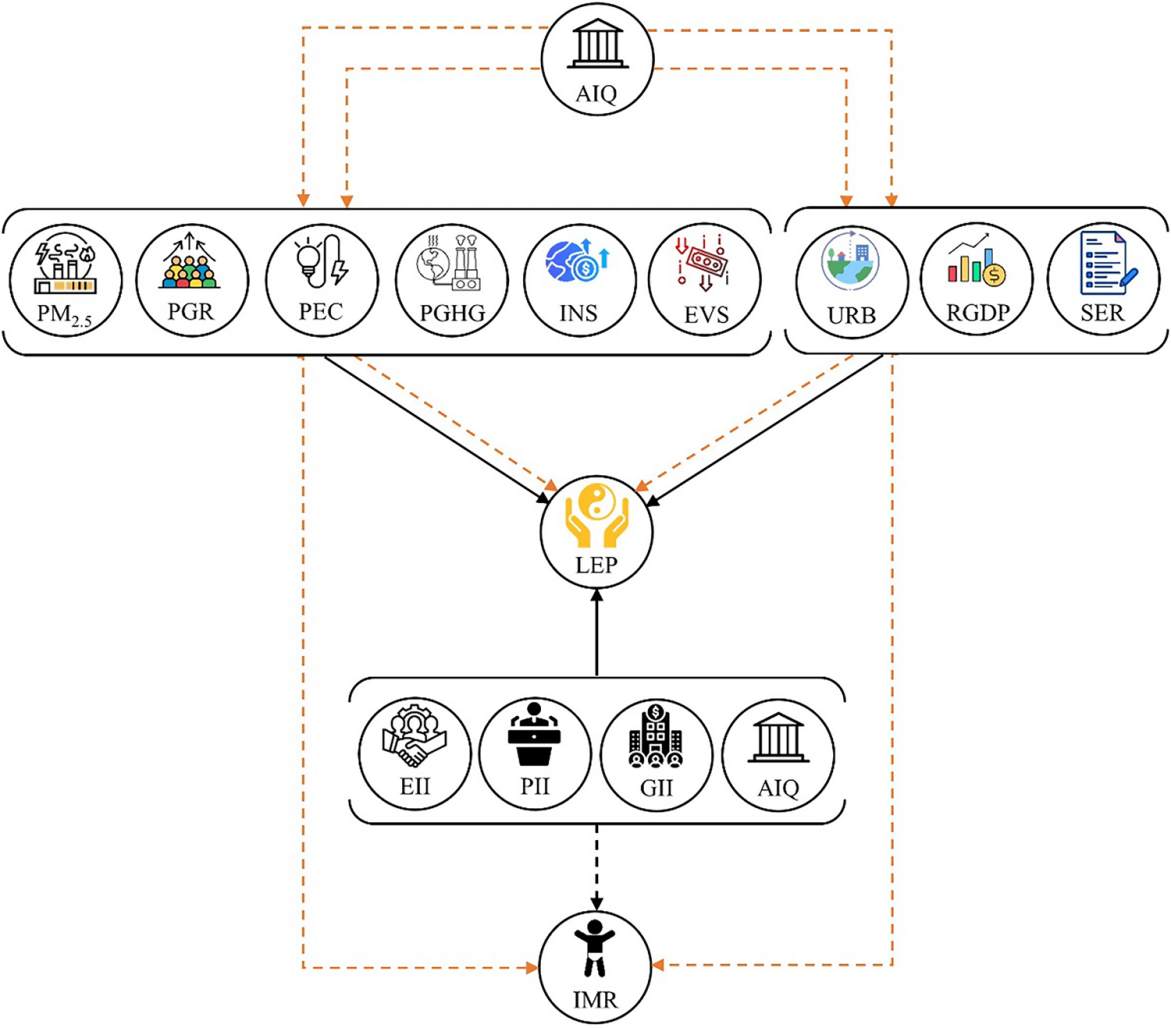
Summary of results. Notes: Solid lines, black dashed lines, and orange dashed lines denote positive effects, negative effects, and moderating effects, respectively. Source: Authors’ creation.

For simplification, we summarize the results of the effects of air pollution, control variables, and exogenous variables on life expectancy and infant mortality rates in Fig. 8.

## Conclusions

With reference to the empirical assumptions and hitherto gaps in the literature, the present study developed new conjectures regarding how air pollution affects health outcome indicators. These conjectures are further motivated by assessing the influence of air pollution on the subject in the presence of exogenous variables using an extended form of the health production function framework (EHPF). The EHPF is then augmented with external shock predictors such as inflationary and economic volatility shocks, while considering the spillover and moderating role of institutional quality on the nexus between the HPF’s endogenous, exogenous, and health outcome indicators. To support a precise evaluation of the hypotheses and highlight policy areas where interventions are sought, the study constructed new variables. The inflationary and economic volatility shock variables have been constructed using the generalized autoregressive conditional heteroskedasticity approach and the CPI-based inflation and growth rate datapoints. It also constructed an aggregate institutional quality index, and sector-specific dimensional indices focusing on economic, political, and institutional governance perspectives. The collected data from various reliable sources ranged from 2000 to 2020, focusing on the top 20 most polluted countries. Furthermore, corresponding to the results of the preliminary panel data analysis, the empirical models that involved three baseline equations have been computed using the two-step Sys-GMM technique, and their validity has been verified through the DOLS and FMOLS methods.

The results indicated that PM_2.5_ concentration is significant to decrease life expectancy by 3.69 years and increase contemporary infant mortality rates by 0.294% across the panel that are over the global average. Other augmented EHPF endogenous factors were also found statistically significant to influence both life expectancy and infant mortality rates. Urbanization, real per capita GDP, and school enrollment rate were observed to improve life expectancy and decrease infant mortality rates, while population growth rate, per capita greenhouse gas emissions, and per capita energy consumption were found to shorten life expectancy and increase infant mortality rates. Further, the findings revealed that inflationary shocks and economic volatility shocks are significant to negatively impact health outcomes. The loss of years in life expectancy and rise in infant mortality rates attributed to inflationary shocks are based on swift uprises in the general prices of goods and services, weakening the population’s financial ability to afford standard-quality food and access required healthcare services. Moreover, the loss of years in life expectancy and rises in infant mortality rates caused by economic volatility shocks are attributed to the loss of employment, risk-averse consumption, and holdbacks in investments due to monetary overhangs. This, in turn, leads the population to long-term exposure to healthcare limitations across the panel. Nonetheless, institutional quality index has been found to improve health outcomes. More precise results were found by evaluating the effects of dimensional-specific indices on health outcome indicators (say, life expectancy and infant mortality rates). They revealed that economic institutions have comparatively higher effects on the subject than those of political and institutional governance indices. Finally, we examined the moderating role of institutional quality on health outcomes to observe whether cross-sector favors are required to improve health outcomes. The results indicated that institutional quality does not only play an effective role in reducing the negative impact of PM_2.5_ concentration on health outcomes; it also offsets the influence of external shocks on the subject and improves the relationships between health outcomes and the remaining variables. The overall findings of the present study highlight specific implications from policy and research perspectives that are explicitly discussed as follows:

### Policy implications

These results clearly imply that policymakers in all other domains must regularly take health outcomes—benefits, risks, and health-related expenses—into account to achieve health equity, health gains, and the attainment of health as an essential human right. Crucially, obtaining improved health outcomes by receiving favors from other government sectors to mitigate the risk of air pollution is not the only option for cross-sector attempts for better and more transparent health outcomes. Instead, it involves the health sector working in tandem with other sectors to design and conduct policies, programs, and initiatives within their own purview in a way that maximizes mutual advantages for all parties. Thus, it calls for stronger commitment and the realization of enhanced institutional quality at both macro- and sector-specific levels to mitigate the negative impact of external shocks on the subject. To be more specific, the results highlight that both governance and political institutions are more critical to addressing air pollution challenges in the countries under review. Substantial focus is sought to improve the quality of economic institutions and political governance to rectify the contemporary air pollution challenges that expose risk to the population’s health outcomes.

### Research implications

The available literature on the complexity of the present topic is insufficient to highlight other hidden aspects of health outcome openness to external shocks and internal deficiencies. The present article has only addressed a chunk of unattended stock. Further studies are required to enhance the contemporary body of knowledge to assist policymakers in taking more specific actions with regards to the impact of air pollution on health outcomes in different economic contexts. Future studies may follow a similar framework while extending their focus on food insecurity, income inequality, gender-based employment, poverty, and health insurance aspects.

### Study limitations

We drew our conclusions based on the specific empirical tests and our reliance on the available datasets from reliable sources for the countries under review. Since economic structures, governance settings, and the degree of openness of other panels or countries may substantively vary from those used in this article, generalizations of the presented results should be used with caution. Although we tried to minimize the limitations to a possible low level, the availability of higher data frequencies that assure more accuracy and lower variances in estimations has been the key limitation of this study. Future studies may overcome this empirical challenge if high-frequency datasets are made available.

## Data and materials

The datasets analyzed during the current study are not publicly available due to institution’s restrictions but will be available from the corresponding author on reasonable request.

## References

[1] Dong D, Wang J (2023). Air pollution as a substantial threat to the improvement of agricultural total factor productivity: Global evidence. Environ. Int..

[2] WHO, WHO ambient air quality database, 2022 update: status report (2022). Available: https://www.who.int/publications/i/item/9789240047693.

[3] Perera F (2018). Pollution from fossil-fuel combustion is the leading environmental threat to global pediatric health and equity: Solutions exist. Int. J. Environ. Res. Public Health.

[4] Rentschler J, Leonova N (2023). Global air pollution exposure and poverty. Nat. Commun..

[5] SGA, Exposure to Air Pollution Reduces Life Expectancy. State of Global Air (2023). Available: https://www.stateofglobalair.org/health/life-expectancy#:~:text=Learn More-SIGNIFICANT IMPACTS, 8 months on average worldwide.

[6] G. B. of Disease, Global Health Data Exchange (GHDx) (2023). Available: https://www.healthdata.org/data-tools-practices/data-sources.

[7] Pandey A (2021). Health and economic impact of air pollution in the states of India: the Global Burden of Disease Study 2019. Lancet Planet. Heal..

[8] Nguyen NM, Luu NH, Hoang A, Nguyen MTN (2023). Environmental impacts of green bonds in cross-countries analysis: A moderating effect of institutional quality. J. Financ. Econ. Policy.

[9] Hu Y, Yao M, Liu Y, Zhao B (2020). Personal exposure to ambient PM2.5, PM10, O3, NO2, and SO2 for different populations in 31 Chinese provinces. Environ. Int..

[10] Zhao S, Shi A, An H, Zhou H, Hu F (2023). Does the low-carbon city pilot contribute to the blue sky defense? Evidence from China. Environ. Sci. Pollut. Res..

[11] Liu Y, Cao G, Zhao N (2020). Integrate machine learning and geostatistics for high-resolution mapping of ground-level PM2.5 concentrations. Spat. Anal. Air Pollut. Its Appl. Public Heal..

[12] Xing YF, Xu YH, Shi MH, Lian YX (2016). The impact of PM2.5 on the human respiratory system. J. Thorac. Dis..

[13] Paul AR, Jain A, Saha SC (2022). Exposure assessment of air pollution in lungs. Atmosphere (Basel)..

[14] Zhong S, Yu Z, Zhu W (2019). Study of the effects of air pollutants on human health based on baidu indices of disease symptoms and air quality monitoring data in Beijing, China. Int. J. Environ. Res. Public Health.

[15] EPA, Health and Environmental Effects of Particulate Pollutants, in *Fine Particulate Pollution* 9–20 (1979).

[16] Apte JS, Brauer M, Cohen AJ, Ezzati M, Pope CA (2018). Ambient PM2.5 reduces global and regional life expectancy. Environ. Sci. Technol. Lett..

[17] Broome RA, Fann N, Cristina TJN, Fulcher C, Duc H, Morgan GG (2015). The health benefits of reducing air pollution in Sydney, Australia. Environ. Res..

[18] Andreão WL, Albuquerque TTA, Kumar P (2018). Excess deaths associated with fine particulate matter in Brazilian cities. Atmos. Environ..

[19] Cui M (2018). Emissions and characteristics of particulate matter from rainforest burning in the Southeast Asia. Atmos. Environ..

[20] Linh Nguyen TN, Pimonsree S, Prueksakorn K, Bich Thao PT, Vongruang P (2022). Public health and economic impact assessment of PM2.5 from open biomass burning over countries in mainland Southeast Asia during the smog episode. Atmos. Pollut. Res..

[21] Pimonsree S, Vongruang P, Sumitsawan S (2018). Modified biomass burning emission in modeling system with fire radiative power: Simulation of particulate matter in Mainland Southeast Asia during smog episode. Atmos. Pollut. Res..

[22] Shang K, Xu L, Liu X, Yin Z, Liu Z, Li X, Zheng W (2023). Study of urban heat island effect in hangzhou metropolitan area based on SW-TES algorithm and image dichotomous model. Sage Open.

[23] Chen XH, Tee K, Elnahass M, Ahmed R (2023). Assessing the environmental impacts of renewable energy sources: A case study on air pollution and carbon emissions in China. J. Environ. Manage..

[24] Lago-Peñas S, Cantarero-Prieto D, Blázquez-Fernández C (2013). On the relationship between GDP and health care expenditure: A new look. Econ. Model..

[25] Thoa NTM, Thanh NX, Chuc NTK, Lindholm L (2013). The impact of economic growth on health care utilization: A longitudinal study in rural Vietnam. Int. J. Equity Health.

[26] Jakovljevic M (2020). Real GDP growth rates and healthcare spending - Comparison between the G7 and the EM7 countries. Global. Health.

[27] Niu G, Melenberg B (2014). Trends in mortality decrease and economic growth. Demography.

[28] Fan Y, Fang M, Zhang X, Yu Y (2023). Will the economic growth benefit public health? Health vulnerability, urbanization and COVID-19 in the USA. Ann. Reg. Sci..

[29] Swift R (2011). The relationship between health and GDP in OECD countries in the very long run. Health Econ..

[30] French D (2012). Causation between health and income: A need to panic. Empir. Econ..

[31] Cohen AK, Syme SL (2013). Education: A missed opportunity for public health intervention. Am. J. Public Health.

[32] Baker DP, Leon J, Smith Greenaway EG, Collins J, Movit M (2011). The education effect on population health: A reassessment. Popul. Dev. Rev..

[33] Albert C, Davia MA (2011). Education is a key determinant of health in Europe: A comparative analysis of 11 countries. Health Promot. Int..

[34] Raghupathi V, Raghupathi W (2020). The influence of education on health: An empirical assessment of OECD countries for the period 1995–2015. Arch. Public Heal..

[35] Torres C, Canudas-Romo V, Oeppen J (2019). The contribution of urbanization to changes in life expectancy in Scotland, 1861–1910. Popul. Stud. (NY).

[36] Jemiluyi OO (2021). Urbanization and child health outcomes in Nigeria. J. Popul. Soc. Stud..

[37] Shao Q, Tao R, Luca MM (2022). The effect of urbanization on health care expenditure: Evidence from China. Front. Public Heal..

[38] Jiang TB, Deng ZW, Zhi YP, Cheng H, Gao Q (2021). The effect of urbanization on population health: Evidence from China. Front. public Heal..

[39] Wang F, Liu S, Chen T, Zhang H, Zhang Y, Bai X (2023). How urbanization affects residents’ health risks: Evidence from China. Environ. Sci. Pollut. Res..

[40] Conceicao GMS, Miraglia SGEK, Kishi HS, Saldiva PHN, Singer JM (2001). Air pollution and child mortality: A time-series study in Sao Paulo, Brazil. Environ. Health Perspect..

[41] Coyle D (2003). Impact of particulate air pollution on quality-adjusted life expectancy in Canada. J. Toxicol. Environ. Heal. - Part A.

[42] Yin P (2020). The effect of air pollution on deaths, disease burden, and life expectancy across China and its provinces, 1990–2017: An analysis for the Global Burden of Disease Study 2017. Lancet Planet. Heal..

[43] Anwar A, Ullah I, Younis M, Flahault A (2021). Impact of air pollution (PM2.5) on child mortality: Evidence from sixteen Asian countries. Int. J. Environ. Res. Public Health.

[44] Tsai SS, Chen CC, Yang CY (2022). The impacts of reduction in ambient fine particulate (PM2.5) air pollution on life expectancy in Taiwan. J. Toxicol. Environ. Heal—Part A Curr. Issues.

[45] Erdoğan S, Yıldırım DÇ, Gedikli A (2019). The relationship between CO_2_ emissions and health indicators: The case of Turkey. Econom. Lett..

[46] Chersich MF, Wright CY, Venter F, Rees H, Scorgie F, Erasmus B (2018). Impacts of climate change on health and wellbeing in South Africa. Int. J. Environ. Res. Public Health.

[47] Hopkins S (2006). Economic stability and health status: Evidence from East Asia before and after the 1990s economic crisis. Health Policy (New York)..

[48] Astell-Burt T, Feng X (2008). Health and the 2008 Economic Recession: Evidence from the United Kingdom”. PLoS One.

[49] Tiwari, S., Zaman, H., The impact of economic shocks on global undernourishment, *World*, Policy Res, no. February, 6279­6287 (2010). Available: http://ssrn.com/paper=1559733.

[50] Bao W, Tao R, Afzal A, Dördüncü H (2022). Real estate prices, inflation, and health outcomes: Evidence from developed economies. Front. Public Heal..

[51] Kawachi I, Kyriopoulos I, Vandoros S (2023). Economic uncertainty and cardiovascular disease mortality. Heal. Econ. (United Kingdom).

[52] Makuta I, Ohare B (2015). Quality of governance, public spending on health and health status in Sub Saharan Africa: A panel data regression analysis. BMC Public Health.

[53] Dhrifi A (2020). Public health expenditure and child mortality: Does institutional quality matter?. J. Knowl. Econ..

[54] De Luca G, Lisi D, Martorana M, Siciliani L (2021). Does higher institutional quality improve the appropriateness of healthcare provision?. J. Public Econ..

[55] Ibukun CO (2021). The role of governance in the health expenditure–health outcomes nexus: insights from West Africa. Int. J. Soc. Econ..

[56] Sharma A, Sharma V, Tokas S (2022). Institutional quality and health outcomes: Evidence from the EU countries. Econom. Bus. Lett..

[57] Beyene SD (2023). The impact of food insecurity on health outcomes: Empirical evidence from sub-Saharan African countries. BMC Public Health.

[58] Banerjee S, Radak T, Khubchandani J, Dunn P (2021). Food insecurity and mortality in American adults: Results from the NHANES-linked mortality study. Health Promot. Pract..

[59] Pole JD, Grossman M (1974). The demand for health: A theoretical and empirical investigation. J. R. Stat. Soc. Ser. A.

[60] Auster R, Leveson I, Sarachek D (1969). The production of health, an exploratory study. J. Hum. Resour..

[61] Cobb C, Douglas P (1928). A theory of production. Am. Econ. Assoc..

[62] Bayati M, Akbarian R, Kavosi Z (2013). Determinants of life expectancy in eastern Mediterranean region: A health production function. Int. J. Heal. Policy Manag..

[63] Fayissa B, Gutema P (2005). Estimating a health production function for Sub-Saharan Africa (SSA). Appl. Econ..

[64] Majumder SC, Dilanchiev A, Rahman H (2022). Examination of a health production function: Evidence from South Asian countries. Southeast Asian J. Econ..

[65] Manisalidis I, Stavropoulou E, Stavropoulos A, Bezirtzoglou E (2020). Environmental and health impacts of air pollution: A review. Front. Public Heal..

[66] Noël C, Vanroelen C, Gadeyne S (2021). Qualitative research about public health risk perceptions on ambient air pollution. A review study. SSM—Popul Heal.

[67] IQAIR, World’s most polluted countries & regions. (2022). Available: https://www.iqair.com/world-most-polluted-countries.

[68] Saito Y, Robine JM, Crimmins EM (2014). The methods and materials of health expectancy. Stat. J. IAOS.

[69] Reidpath DD, Allotey P (2003). Infant mortality rate as an indicator of population health. J. Epidemiol. Commun Health.

[70] Gonzalez RM, Gilleskie D (2017). Infant mortality rate as a measure of a Country’s health: A robust method to improve reliability and comparability. Demography.

[71] Wang X, Yang H, Duan Z, Pan J (2018). Spatial accessibility of primary health care in China: A case study in Sichuan Province. Soc. Sci. Med..

[72] Wu IP, Liao SL, Lai SH, Wong KS (2022). The respiratory impacts of air pollution in children: Global and domestic (Taiwan) situation. Biomed. J..

[73] Siddiqua A, Hahladakis JN, Al-Attiya WAKA (2022). An overview of the environmental pollution and health effects associated with waste landfilling and open dumping. Environ. Sci. Pollut. Res..

[74] Luy M, Zannella M, Wegner-Siegmundt C, Minagawa Y, Lutz W, Caselli G (2019). The impact of increasing education levels on rising life expectancy: a decomposition analysis for Italy, Denmark, and the USA. Genus.

[75] Malamud O, Mitrut A, Pop-Eleches C (2021). The effect of education on mortality and health: Evidence from a schooling expansion in Romania. J. Hum. Resour..

[76] Zhang L, You S, Zhang M, Zhang S, Yi S, Zhou B (2022). The effects of urbanization on air pollution based on a spatial perspective: Evidence from China. Front. Environ. Sci..

[77] Stefko R, Gavurova B, Kelemen M, Rigelsky M, Ivankova V (2021). Relationships between renewable energy and the prevalence of morbidity in the countries of the european union: A panel regression approach. Int. J. Environ. Res. Public Health.

[78] Avashia V, Garg A, Dholakia H (2021). Understanding temperature related health risk in context of urban land use changes. Landsc. Urban Plan..

[79] Gavurova B, Rigelsky M, Ivankova V (2021). Greenhouse gas emissions and health in the countries of the European Union. Front. Public Heal..

[80] Karanikolos M, Heino P, McKee M, Stuckler D, Legido-Quigley H (2016). Effects of the global financial crisis on health in high-income OECD countries: A narrative review. Int. J. Heal. Serv..

[81] Haileamlak A (2021). The impact of COVID-19 on health and health systems. Ethiop. J. Health Sci..

[82] Frawley T (2021). The impact of COVID-19 on health systems, mental health and the potential for nursing. Irish J. Psychol. Med..

[83] Hameed MA, Rahman MM, Khanam R (2023). The health consequences of civil wars: evidence from Afghanistan. BMC Public Health.

[84] Lutz C, Mazzarino A (2020). War and health: The medical consequences of the wars in Iraq and Afghanistan. New York Univ. Press.

[85] Ipinnimo TM, Adeniyi IO, Osuntuyi OMM, Ehizibue PE, Ipinnimo MT, Omotoso AA (2023). The unspotted impact of global inflation and economic crisis on the Nigerian healthcare system. Asia Pacif. J. Health Manag..

[86] Abdullah MN, Shamsher R, Chowdhury NA (2012). Consequences and causes of inflation: A study in the context of Bangladesh. World J. Soc. Sci..

[87] Fadul N, Hussein ME, Fadul AA (2021). Re-opening Sudan: The balance between maintaining daily living and avoiding the next peak of COVID-19. Curr. Trop. Med. Reports.

[88] Gokbulut RI, Pekkaya M (2014). Estimating and forecasting volatility of financial markets using asymmetric GARCH models: An application on Turkish financial markets. Int. J. Econ. Financ..

[89] Abaidoo R, Agyapong EK (2022). Financial development and institutional quality among emerging economies. J. Econ. Dev..

[90] Azimi MM, Mohammad Naim R (2023). Impact of institutional quality on ecological footprint: New insights from G20 countries. J. Clean. Prod..

[91] Cai R (2019). Violence against health care workers in China, 2013–2016: Evidence from the national judgment documents. Hum. Resour. Health.

[92] Dasgupta S, Hamilton K, Pandey KD, Wheeler D (2006). Environment During growth: Accounting for governance and vulnerability. World Dev..

[93] Sarma, M., Index of financial inclusion—A measure of financial sector inclusiveness, *Berlin Work. Pap. Money, Financ. Trade Dev.*, 24(8), 472–476 (2012). Accessed: Jun. 12, 2022. Available: https://finance-and-trade.htw-berlin.de/fileadmin/HTW/Forschung/Money_Finance_Trade_Development/working_paper_series/wp_07_2012_Sarma_Index-of-Financial-Inclusion.pdf.

[94] Park, C., Mercado, R. V., Financial inclusion, poverty, and income inequality in developing Asia. ADB Economics Working pepers series. *ADB Economics Working Paper Series 426. Manila, Philippines*, (2015).

[95] Azimi MN, Rahman MM, Nghiem S (2023). A global perspective on the governance-health nexus. BMC Health Serv. Res..

[96] Kaufmann, D., Kraay, A., Worldwide Governance Indicators (WGI) Project, *World Bank* (2018).

[97] Azimi MN (2022). Revisiting the governance-growth nexus: Evidence from the world’s largest economies. Cogent Econ. Financ..

[98] Azimi MN, Shafiq MM (2020). Hypothesizing directional causality between the governance indicators and economic growth: the case of Afghanistan. Future Bus. J..

[99] Omar MA, Inaba K (2020). Does financial inclusion reduce poverty and income inequality in developing countries? A panel data analysis. J. Econ. Struct..

[100] World Bank, World development indicators | data, *World Development Indicators* (2023). https://data.worldbank.org/indicator.

[101] W. U. in St. Louis, Global PM25-V5GL03-Annual-1998–2021-wThresFrac.csv. (2023). Available: https://wustl.app.box.com/s/7wjvfr0fn5lot67g5j8cxqq0u5afdwln.

[102] Ritchie, H., Roser, M., Renewable Energy—Our World in Data, *Our World in Data* (2020). Available: https://ourworldindata.org/renewable-energy.

[103] Drukker DM (2003). Testing for serial correlation in linear panel-data models. Stata J. Promot. Commun. Stat. Stata.

[104] Smith RJ, Hsiao C (1988). Analysis of panel data. Economica.

[105] Pesaran, M. H., General diagnostic tests for cross section dependence in panels. *Univ. Cambridge, Fac. Econ. Cambridge Work. Pap. Econ. No. 0435*, 1–37 (2004).

[106] O’Brien RM (2007). A caution regarding rules of thumb for variance inflation factors. Qual. Quant..

[107] Kiviet JF (1995). On bias, inconsistency, and efficiency of various estimators in dynamic panel data models. J. Econom..

[108] Chudik A, Pesaran MH (2015). Common correlated effects estimation of heterogeneous dynamic panel data models with weakly exogenous regressors. J. Econom..

[109] Arellano M, Bond S (1991). Some tests of specification for panel data. Rev. Econ. Stud..

[110] Bound J, Jaeger DA, Baker RM (1995). Problems with instrumental variables estimation when the correlation between the instruments and the endogeneous explanatory variable is weak. J. Am. Stat. Assoc..

[111] Hwang J, Sun Y (2018). Should we go one step further? An accurate comparison of one-step and two-step procedures in a generalized method of moments framework. J. Econom..

[112] Sargan, J. D., *Testing for misspecification after estimating using instrumental variables* (1988).

[113] Lyu Y, Ali SA, Yin W, Kouser R (2022). Energy transition, sustainable development opportunities, and carbon emissions mitigation: is the developed world converging toward SDGs-2030?. Front. Environ. Sci..

[114] Muye IM, Muye IY (2017). Testing for causality among globalization, institution and financial development: Further evidence from three economic blocs. Borsa Istanbul Rev..

[115] Pesaran MH (2007). A simple panel unit root test in the presence of cross-section dependence. J. Appl. Econom..

[116] Im KS, Pesaran MH, Shin Y (2003). Testing for unit roots in heterogeneous panels. J. Econom..

[117] Levin A, Lin CF, Chu CSJ (2002). Unit root tests in panel data: Asymptotic and finite-sample properties. J. Econom..

[118] Westerlund J (2007). Testing for error correction in panel data. Oxf. Bull. Econ. Stat..

[119] Röösli M, Künzli N, Braun-Fahrländer C, Egger M (2005). Years of life lost attributable to air pollution in Switzerland: Dynamic exposure-response model. Int. J. Epidemiol..

[120] State of Global Air, Impact of Air Pollution on Life Expectancy (2019). Available: https://www.stateofglobalair.org/health/life-expectancy.

[121] Lelieveld J, Pozzer A, Pöschl U, Fnais M, Haines A, Münzel T (2020). Loss of life expectancy from air pollution compared to other risk factors: A worldwide perspective. Cardiovasc. Res..

[122] Folinsbee LJ (1993). Human health effects of air pollution. Environ. Health Perspect..

[123] Pope CA, Dockery DW (2006). Health effects of fine particulate air pollution: Lines that connect. J. Air Waste Manag. Assoc..

[124] Kampa M, Castanas E (2008). Human health effects of air pollution. Environ. Pollut..

[125] Burnett RT (2014). An integrated risk function for estimating the global burden of disease attributable to ambient fine particulate matter exposure. Environ. Health Perspect..

[126] Guo Y, Li S, Tawatsupa B, Punnasiri K, Jaakkola JJK, Williams G (2014). The association between air pollution and mortality in Thailand. Sci. Rep..

[127] Ghorani-Azam A, Riahi-Zanjani B, Balali-Mood M (2016). Effects of air pollution on human health and practical measures for prevention in Iran. J. Res. Med. Sci..

[128] Miller L, Xu X (2018). Ambient PM2.5 human health effects-findings in China and research directions. Atmosphere (Basel)..

[129] Almetwally AA, Bin-Jumah M, Allam AA (2020). Ambient air pollution and its influence on human health and welfare: An overview. Environ. Sci. Pollut. Res..

[130] Wang L (2021). Effects of PM2.5 exposure on reproductive system and its mechanisms. Chemosphere.

[131] Esmaeili P, Balsalobre Lorente D, Anwar A (2023). Revisiting the environmental Kuznetz curve and pollution haven hypothesis in N-11 economies: Fresh evidence from panel quantile regression. Environ. Res..

[132] Duodu E, Mpuure DMN (2023). International trade and environmental pollution in sub-Saharan Africa: Do exports and imports matter?. Environ. Sci. Pollut. Res..

[133] Mahalik MK, Le T-H, Le H-C, Mallick H (2022). How do sources of carbon dioxide emissions affect life expectancy? Insights from 68 developing and emerging economies. World Dev. Sustain..

[134] Ansarinasab M, Bidmal N (2022). The impact of environmental pollutants emission (carbon dioxide) on life expectancy of men and women in Iran. Iran. J. Heal. Environ..

[135] Bhutto, N. A., Chang, B. H., Adeel, S., Seelro, A. D., & Qureshi, M. U. Global warming economic development and their impact on the life expectancy An empirical evidence from Pakistan. *Stud. Appl. Econ*. (2023). 10.25115/sae.v41i1.7875.

[136] Schaefer KE (1982). Effects of increased ambient CO_2_ levels on human and animal health. Experientia.

[137] Rice SA (2004). Human health risk assessment of CO_2_: survivors of acute high-level exposure and population sensitive to prolonged low-level exposure. Third Annu. Conf. Carbon Sequestration.

[138] Zhang Z, Zhao M, Zhang Y, Feng Y (2023). How does urbanization affect public health? New evidence from 175 countries worldwide. Front. Public Heal..

[139] Fan Y, Fang C, Zhang Q (2019). Coupling coordinated development between social economy and ecological environment in Chinese provincial capital cities-assessment and policy implications. J. Clean. Prod..

[140] Wang Q (2018). Urbanization and global health: The role of air pollution. Iran. J. Public Health.

[141] Gong P (2012). Urbanisation and health in China. Lancet.

[142] Ahmad N, Raid M, Alzyadat J, Alhawal H (2023). Impact of urbanization and income inequality on life expectancy of male and female in South Asian countries: A moderating role of health expenditures. Humanit. Soc. Sci. Commun..

[143] Eckert S, Kohler S (2014). Urbanization and health in developing countries: a systematic review. World Health Popul..

[144] Miladinov G (2020). Socioeconomic development and life expectancy relationship: evidence from the EU accession candidate countries. Genus.

[145] Majeed MT, Ozturk I (2020). Environmental degradation and population health outcomes: A global panel data analysis. Environ. Sci. Pollut. Res..

[146] Boz C, Ozsarı SH (2020). The causes of aging and relationship between aging and health expenditure: An econometric causality analysis for Turkey. Int. J. Health Plann. Manage..

[147] Chaabouni S, Zghidi N, Ben Mbarek M (2016). On the causal dynamics between CO_2_ emissions, health expenditures and economic growth. Sustain. Cities Soc..

[148] Kibirige JS (1997). Population growth, poverty and health. Soc. Sci. Med..

[149] Marques AC, Fuinhas JA, Leal PA (2018). The impact of economic growth on CO_2_ emissions in Australia: The environmental Kuznets curve and the decoupling index. Environ. Sci. Pollut. Res..

[150] Lange S, Vollmer S (2017). The effect of economic development on population health: A review of the empirical evidence. Br. Med. Bull..

[151] Luo W, Xie Y (2020). Economic growth, income inequality and life expectancy in China. Soc. Sci. Med..

[152] Niu XT, Yang YC, Wang YC (2021). Does the economic growth improve public health? A cross-regional heterogeneous study in China. Front. Public Heal..

[153] Cutler D, Deaton A, Lleras-Muney A (2006). The determinants of mortality. J. Econ. Perspect..

[154] Kaur A (2023). Health status, government health expenditure and economic growth nexus in India: A Toda-Yamamoto causality approach. Arthaniti J. Econ. Theory Pract..

[155] Polcyn J, Voumik LC, Ridwan M, Ray S, Vovk V (2023). Evaluating the influences of health expenditure, energy consumption, and environmental pollution on life expectancy in Asia. Int. J. Environ. Res. Public Health.

[156] Taghizadeh-Hesary F, Rasoulinezhad E, Yoshino N (2019). Energy and food security: Linkages through price volatility. Energy Policy.

[157] Heflin C, Darolia R, Kukla-Acevedo S (2022). Exposure to food insecurity during adolescence and educational attainment. Soc. Probl..

[158] Ali A, Ahmad K (2014). The impact of socio-economic factors on life expectancy in sultanate of Oman: An empirical analysis. Middle-East J. Sci. Res..

[159] Hansen CW, Strulik H (2017). Life expectancy and education: Evidence from the cardiovascular revolution. J. Econ. Growth.

[160] Azam M, Haroon Hafeez M, Khan F, Abdullah H (2019). Impacts of education and life expectancy on economic growth: Panel data evidence from developing economies Pakistan. J. Soc. Sci..

[161] Rahman MM, Alam K (2023). The role of socio-economic and female indicators on child mortality rate in Bangladesh: A time series analysis. Omega (United States).

[162] Bechtel G (2022). Human development and life expectancy perfectly predict inflation. Open J. Soc. Sci..

[163] McDaid D (2013). Health protection in times of economic crisis: Challenges and opportunities for Europe. J. Public Health Policy.

[164] Glonti K (2015). A systematic review on health resilience to economic crises. PLoS One.

[165] Ruhm CJ (2016). Health effects of economic crises. Heal. Econ. (United Kingdom).

[166] Antonova L, Bucher-Koenen T, Mazzonna F (2017). Long-term health consequences of recessions during working years. Soc. Sci. Med..

[167] Ahmad W, Ullah S, Ozturk I, Majeed MT (2021). Does inflation instability affect environmental pollution? Fresh evidence from Asian economies. Energy Environ..

[168] Prędkiewicz P, Bem A, Siedlecki R, Kowalska M, Robakowska M (2022). An impact of economic slowdown on health. New evidence from 21 European countries. BMC Public Health.

[169] Hadipour A, Delavari S, Bayati M (2023). What is the role of institutional quality in health outcomes? A panel data analysis on 158 countries from 2001–2020. Heliyon.

[170] Rahman MM, Alam K (2022). Life expectancy in the ANZUS-BENELUX countries: The role of renewable energy, environmental pollution, economic growth and good governance. Renew. Energy.

[171] Jafari F, Hajinabi K, Jahangiri K, Riahi L (2018). An analysis of good governance in the health system. J. Clin. Res. Paramed. Sci..

[172] Al-Q-Bousmah M, Ventelou B, Abu-Zaineh M (2016). Medicine and democracy: The importance of institutional quality in the relationship between health expenditure and health outcomes in the MENA region. Health Policy (New York)..

[173] Brinkerhoff DW, Bossert TJ (2014). Health governance: Principal-agent linkages and health system strengthening. Health Policy Planning.

[174] Kaini BK (2013). Healthcare governance for accountability and transparency. J. Nepal Health Res. Counc..

[175] Kirigia JM, Kirigia DG (2011). The essence of governance in health development. Int. Arch. Med..

[176] Rehmat, S., Majeed, M. T., Zainab, A., Health outcomes of institutional quality: A cross country analysis. *Empir. Econ. Rev.* 3(1), 19–40 (2020). Available: https://ojs.umt.edu.pk/index.php/eer.

[177] Ibrahim MH, Law SH (2016). Institutional quality and CO_2_ emission–trade relations: Evidence from Sub-Saharan Africa. South African J. Econ..

[178] Salman M, Long X, Dauda L, Mensah CN (2019). The impact of institutional quality on economic growth and carbon emissions: Evidence from Indonesia, South Korea and Thailand. J. Clean. Prod..

[179] Khan M, Rana AT (2021). Institutional quality and CO_2_ emission–output relations: The case of Asian countries. J. Environ. Manage..

[180] Haldar A, Sethi N (2021). Effect of institutional quality and renewable energy consumption on CO_2_ emissions−an empirical investigation for developing countries. Environ. Sci. Pollut. Res..

[181] Yuan B, Li C, Yin H, Zeng M (2022). Green innovation and China’s CO_2_ emissions–the moderating effect of institutional quality. J. Environ. Plan. Manag..

[182] Adams S, Klobodu EKM (2017). Urbanization, democracy, bureaucratic quality, and environmental degradation. J. Policy Model..

[183] Sun Y, Tian W, Mehmood U, Zhang X, Tariq S (2023). How do natural resources, urbanization, and institutional quality meet with ecological footprints in the presence of income inequality and human capital in the next eleven countries?. Resour. Policy.

[184] Azam M, Liu L, Ahmad N (2021). Impact of institutional quality on environment and energy consumption: evidence from developing world. Environ. Dev. Sustain..

[185] Nawaz S, Iqbal N, Khan MA (2014). The impact of institutional quality on economic growth: Panel evidence. Pak. Dev. Rev..

[186] Nirola N, Sahu S (2019). The interactive impact of government size and quality of institutions on economic growth- evidence from the states of India. Heliyon.

[187] Wandeda DO, Masai W, Nyandemo SM (2021). Institutional quality and economic growth: evidence from Sub-Saharan Africa countries. African J. Econ. Rev..

[188] Opeloyeru OS, Faronbi TO, Raifu IA (2023). The role of institutional quality in health expenditure-labor force participation Nexus in Africa. J. Knowl. Econ..

[189] Tadadjeu S, Njangang H, Asongu SA, Kamguia B (2023). Natural resources, child mortality and governance quality in African countries. Resour. Policy.

[190] Ouedraogo I, Dianda I, Adeyele IT (2020). Institutional quality and health outcomes in Sub-Saharan Africa. Res. Appl. Econ..

[191] Alimi OY, Ajide KB (2021). The role of institutions in environment–health outcomes Nexus: empirical evidence from sub-Saharan Africa. Econ. Chang. Restruct..

[192] Asgher N, Ur Rehman H, Mumtaz A (2018). Impact of institutional quality, energy prices and financial development on income inequality: Evidence from selected Asian Countries. J. Res. Soc. Pakistan.

[193] Asamoah ME, Adjasi CKD, Alhassan AL (2016). Macroeconomic uncertainty, foreign direct investment and institutional quality: Evidence from Sub-Saharan Africa. Econ. Syst..

